# The Signal in the Extreme: A Systematic Outlier Framework Identifies Discrete Immunometabolic Subtypes in Human and Cellular Models

**DOI:** 10.3390/medsci14010128

**Published:** 2026-03-09

**Authors:** Julio Jesús Garcia-Coste, Karla Aidee Aguayo-Cerón, Judith Espinosa-Raya, Alexis Alejandro García-Rivero, Carina López-Leyva, Rocío Alejandra Gutiérrez-Rojas, Cruz Vargas-De-León, Rodrigo Romero-Nava

**Affiliations:** 1 Laboratorio de Investigación en Genética de Enfermedades Metabólicas, Escuela Superior de Medicina, Instituto Politécnico Nacional, Ciudad de México 11340, Mexico; jjcoste18@gmail.com (J.J.G.-C.); carinalleyva@gmail.com (C.L.-L.); 2 Laboratorio Multidisciplinario en Ciencias Biomédicas, Escuela Superior de Medicina, Instituto Politécnico Nacional, Ciudad de México 11340, Mexico; jespinosara@ipn.mx; 3 Laboratorio de Biología Molecular, Escuela Superior de Medicina, Instituto Politécnico Nacional, Ciudad de México 11340, Mexico; alexciencia9405@gmail.com; 4 Escuela Nacional de Ciencias Biológicas, Instituto Politécnico Nacional, Ciudad de México 11340, Mexico; ross.grojas.22@gmail.com; 5 Laboratorio de Modelación Bioestadística para la Salud, Escuela Superior de Medicina, Instituto Politécnico Nacional, Ciudad de México 11340, Mexico; leoncruz82@yahoo.com.mx; 6 División de Investigación, Hospital Juárez de México, Ciudad de México 07760, Mexico

**Keywords:** outliers, anomaly detection, metabolic syndrome, inflammation, co-expression networks

## Abstract

Background: Conventional omics analysis often treats outliers as noise, yet they may harbor critical biological insights. Objetive: This study proposes a paradigm shift: actively investigating outliers to discover biologically relevant subtypes within metabolic–inflammatory syndromes. Methods: We applied a comprehensive analytical framework for outlier detection based on a multi-algorithm consensus (IQR, MAD, Isolation Forest) to a clinical cohort of diabetic neuropathy (n = 93) and an *in vitro* 3T3-L1 adipocyte model (n = 39). The identified outliers were characterized using robust PCA, co-expression networks, unsupervised clustering, and Random Forest predictive modeling. Results: In the clinical cohort, an outlier subgroup (47.3%) exhibited an extreme immune–metabolic phenotype characterized by hyperactivation of Th1/Th17 pathways (elevated T-bet and IL-17; *p* < 0.001), hypertriglyceridemia, and network reconfiguration (TGFβ and STAT4 hubs). In the cellular model, outlier samples (12.8%) showed autonomous pro-inflammatory behavior characterized by IL-6 overproduction (*p* = 0.002) and IL-10 suppression. Conclusions: Multivariate analysis confirmed spatial segregation of these profiles. Systematic outlier investigation revealed discrete pathophysiological subtypes invisible to mean-focused analyses, demonstrating that extreme values encapsulate potent biological signals. This framework offers a generalizable approach for uncovering clinical heterogeneity and identifying therapeutic targets in complex diseases.

## 1. Introduction

Large-scale gene expression analysis has become a cornerstone of translational biomedical research, enabling the elucidation of molecular mechanisms underlying complex physiological states such as low-grade inflammation, obesity, and metabolic syndrome [1]. Conventionally, statistical rigor in this field has centered on the paradigm of identifying common patterns and significant average differences within study populations. Within this framework, observations that deviate markedly from the expected distribution—commonly referred to as outliers—are frequently treated as a methodological nuisance. Standard analytical practice typically involves detecting and removing such observations to clean datasets and preventing them from distorting the assumptions of parametric statistical models, such as those used in hypothesis testing and regression analyses [2]. This practice, while methodologically sound for the robust estimation of average effects, may come at a significant epistemological cost: the systematic, *a priori* elimination of observations that may encapsulate the most informative and transformative biological signals [3].

This paradox is reflected throughout the history of medical science, which is punctuated by seminal discoveries originating precisely from meticulous attention to atypical observations. A paradigmatic example is the discovery of Kawasaki disease. Dr. Tomisaku Kawasaki identified a series of pediatric cases presenting with an unusual constellation of severe symptoms—prolonged fever, erythema, edema, and desquamation—that did not fit known diagnoses [4].

Subsequent genomic studies have shown that these outliers often harbor polymorphisms in genes of the interleukin-1β (IL-1β) signaling pathway, such as CASP3, and in genes related to B-cell function (BLK, CD40), which may partially explain the unusually strong inflammatory response observed [5]. Similarly, the identification of Hantavirus Pulmonary Syndrome in 1993 arose from the investigation of a cluster of atypical cases of acute and fatal respiratory failure in previously healthy young adults. This epidemiological anomaly ultimately led to the discovery of a new pathogen and disease mechanism [6]. In pharmacogenomics, the investigation of rare but severe cases of rhabdomyolysis in patients treated with statins—an extreme adverse event—led to the identification of loss-of-function variants in the SLCO1B1 gene as their underlying genetic cause, transforming a rare adverse effect into a model for understanding the pharmacogenetics of drug toxicity [7,8].

These historical examples underscore a fundamental methodological truth: an atypical observation, when rigorously investigated, may contain the most potent signal and serve as irrefutable evidence of an alternative etiological mechanism [9].

This reasoning also extends to basic genetic research, where the intentional study of extreme phenotypes has been instrumental in assigning gene function. A prominent natural model is the naked mole-rat (*Heterocephalus glaber*). This rodent constitutes a multiple physiological outlier: it exhibits exceptional longevity (over 30 years) and near-total resistance to spontaneous cancer. Comparative genomic studies have revealed that these extreme phenotypes are associated with distinct molecular mechanisms, including high expression and stability of DNA repair genes (ERCC1) and strict regulation of cell proliferation mediated by the p16INK4a/RB pathway [10,11]. The study of this atypical organism has provided valuable insight into potential mechanisms for modulating aging and oncogenesis in humans.

In a complementary and more direct manner, knockout mouse technology follows the same principle: the artificial generation of an extreme phenotype (the complete absence of a gene’s function) to infer its biological role. The creation of the ob/ob mouse, harboring a mutation in the Leptin (Lep) gene, produced an extreme phenotype of morbid obesity, hyperphagia, and infertility, clearly revealing the central role of this hormone in regulating energy balance [12]. These models demonstrate that outliers constitute powerful tools for causal discovery.

In the context of gene expression, the definition and handling of outliers assume critical importance. According to Hawkins’s seminal definition, an outlier is an observation that deviates so markedly from other observations as to arouse suspicion that it was generated by a different mechanism [13]. As noted by Kacprowicz, such observations may also contain valuable and uncommon information that warrants attention [14]. This mechanism can manifest at multiple levels: a technical artifact, a rare genetic variant with a potent cis-regulatory effect, an extreme physiological state that has not yet been characterized, an exceptional therapeutic response, or a previously uncharacterized biological subpopulation [15]. Reliably distinguishing between noise (error) and signal (discovery) constitutes a central analytical challenge, requiring a robust methodological framework [16].

The problem is compounded by limitations of classical statistical approaches, such as conventional Principal Component Analysis (PCA)—a ubiquitous tool for quality control that is notoriously sensitive to outliers and can be dominated by them, failing to detect subtle multivariate outliers: those that do not display extreme values in any single variable but do in a specific combination of many features [17]. These outliers, which may represent complex and novel biological states, are invisible to univariate techniques and require specialized detection methods, such as the Local Outlier Factor (LOF) or Isolation Forest, that account for the covariance structure and local density of data in high-dimensional spaces.

In recent years, several computational approaches have been developed to identify extreme observations in high-dimensional omics datasets. Methods such as Cancer Outlier Profile Analysis (COPA) were introduced to detect genes exhibiting strong expression in only subsets of tumor samples, enabling the discovery of oncogenic rearrangements that would remain undetected using conventional differential-expression analysis [18]. Similarly, more recent frameworks such as OUTRIDER have been designed to detect aberrantly expressed genes in RNA-sequencing datasets by modeling expression distribution and controlling hidden confounding factors. While these approaches have proven valuable for identifying anomalous expression events, they are primarily focused on statistical detection rather than systematic biological characterization of the detected outliers. Consequently, the integration of anomaly detection with multi-layer biological interpretation remains relatively underdeveloped in translational omics research [19].

Recognizing this potential and these challenges, a recent conceptual framework has been proposed that reframes biomedical discovery as an anomaly detection problem within an augmented intelligence system [20,21]. In this model, computational algorithms screen large, heterogeneous datasets to identify atypical observations or patterns, which are then prioritized for in-depth investigation by human experts, closing the loop with hypothesis generation and validation.

However, the systematic and reproducible application of this approach to multimodal gene expression studies, integrating in silico, in vitro, animal-model, and human data, remains largely unexplored.

The present article proposes and details this approach. We present a comprehensive analytical framework whose objective is not to suppress outliers, but to systematically identify, characterize, and interpret them within datasets related to the pathophysiology of metabolic syndrome and inflammation, transcending the particularities of each study to address general methodological and biological questions: What common biological patterns are revealed when the most extreme gene expressions are systematically analyzed across experimental models? Is it possible to characterize the molecular profile of an outlier and predict its underlying biological or clinical significance? Through this, we aim to demonstrate that a shift in epistemological and methodological perspective—from removal to investigation of extreme values—can accelerate the discovery of novel mechanisms, disease subtype biomarkers, and exceptional therapeutic responses in complex diseases.

Our findings suggest that outliers represent biologically coherent immunometabolic profiles with distinct molecular characteristics, providing a framework for further investigations of potential therapeutic targets.

## 2. Materials and Methods

### 2.1. Study Design and Datasets

Two independent datasets related to gene expression research in the context of low-grade inflammation, obesity, and general components of metabolic syndrome were analyzed:

- In vitro study on 3T3-L1 (ATCC American Type Culture Collection, Manassas, VA, USA, EEUU) adipocytes: We re-analyzed gene expression data from a published study investigating the anti-inflammatory effects of glycine [22]. 3T3-L1 preadipocytes were cultured and differentiated in 6-well plates. Subsequently, siRNA transfections targeting (Dharmacon, Lafayette, CO, USA, EEUU) the Tnfrsf1a (TNFR1a) and Tnfrsf1b (TNFR1b) genes were performed using Lipofectamine 2000 (Invitrogen, Carlsbad, CA, USA, EEUU) in Opti-MEM medium (Gibco/Thermo Fisher Scientific, Waltham, MA, USA, EEUU). Transfected cells were treated with glycine (10 mM) for two short periods (1 and 24 h). The primary objective was to measure the effect of glycine on the gene expression (T4 Oligo-primer, Guanajuato, Mexico) of pro- and anti-inflammatory cytokines (TNF-α, IL-1β, IL-10) and adiponectin (ADIPOQ), through their interaction on these receptors, using quantitative real-time PCR (qRT-PCR) (MyGo Mini, IT-IS Life Science, London, UK). The final data matrix for outlier analysis consisted of gene expression levels (2^−ΔΔCt^ (Livak Method) or normalized values) for multiple cytokines, obtained from biological and technical replicates under different experimental conditions (gene silencing and treatment).
- Clinical study on diabetic neuropathy: We conducted a secondary analysis of baseline data from the master’s degree thesis titled “Evaluación del efecto de la electroacupuntura sobre marcadores genéticos de polarización CD4 en pacientes con neuropatía diabética” by the author López-Leyva (A240351). The original double-blind, randomized controlled trial was designed to evaluate the efficacy of electroacupuncture. For the present study, we utilized the complete baseline dataset from 93 participants (initially stratified into a control group, n = 45, and an electroacupuncture intervention group, n = 48) as a single cohort to discover intrinsic extreme phenotypes. Participants fulfilled diagnostic criteria for type 2 diabetes and clinical evidence of diabetic neuropathy, while individuals with severe systemic disease, active infections, autoimmune disorders, or immunomodulatory therapy were excluded to minimize confounding effects on inflammatory biomarkers. Peripheral blood samples were used to quantify an immunological profile by qRT-PCR (MyGo Mini, IT-IS Life Science, UK), measuring the expression of eleven key mediators (cytokines and transcription factors(T4 Oligo-primer, Mexico)), including IL-17, T-bet, RORγT, STAT3, STAT4, STAT5, STAT6, SOCS1, SOCS3, TGFβ, and TNF-α. Standard clinical and metabolic variables, including glycated hemoglobin (HbA1c, measured by immunoturbidimetry) and a complete lipid profile (measured using standard biochemical assays), were also analyzed.

Prior to the analysis, the datasets were evaluated for completeness. No missing values were present in the variables included in the analytical pipeline; therefore, no imputation procedures were required.

The same outlier detection framework was applied to both datasets, enabling a cross-system methodological evaluation.

### 2.2. Computational Pipeline and Outlier Detection Strategy

The core outlier detection and characterization pipeline was initially developed using an R script in RStudio (v4.5.3). This script served as the primary methodological framework for data processing and analysis. The analytical workflow was subsequently implemented and operationalized in a web application built with a Python (v3.13.2) backend using the FastAPI (v0.104.1) framework with a Uvicorn ASGI server (v0.24.0). The backend leveraged the following libraries for data processing and analysis: *Pandas (v2.2.3)* and *NumPy (v2.2.4)* for data handling; *openpyxl (v3.1.5)* for reading Excel files; *scikit-learn (v1.6.1)* for machine learning algorithms; *Statsmodels (v0.14.5)* for advanced statistical modeling; *NetworkX (v3.4.2)* for network analysis; and *Plotly (v6.0.1)* and *Seaborn (v0.13.2)* for visualization. File validation and management were handled using *python-multipart (v0.0.6)*, *Aiofiles (v24.1.0)*, *chardet (v5.2.0)*, and *Werkzeug (v3.0.1)*. The environment was secured through testing with *Pytest (v9.0.2)*, *pytest-cov (v7.0.0)*, and *pytest-asyncio (v1.3.0)*. The frontend was implemented using HTML, JavaScript, and CSS.

Within the R environment, all described statistical analyses were executed using the following packages: *readxl (v1.4.5)* for basic data manipulation [23]; *stats (v4.5.0)* for statistical tests (Shapiro–Wilk, Mann–Whitney U, chi-square) [24]; *EnvStats (v3.1.0)* for specific outlier tests (Grubbs, Dixon, Rosner) [25]; *robustbase (v0.99-4-1)* [26] and *rrcov (v1.7-7)* [27] for robust models (MAD, robust PCA); *dbscan (v1.2.4)* [28] and *solitude (1.1.3)* [29] for multivariate detection algorithms (LOF, Isolation Forest); *WGCNA (v1.73)* [30] for co-expression network analysis; *randomForest (v4.7-1.2)* [31] and *caret (v7.0-1)* [32] for predictive modeling; and *ggplot2 (v4.0.0)* [33] and *plotly* [34] for visualization.

### 2.3. Data Availability

The processed datasets supporting the conclusions of this article—including the gene expression matrix from the 3T3-L1 adipocyte study and the baseline clinical–immunological data from the diabetic neuropathy cohort—are available in a dedicated repository and can be accessed via the following link: https://drive.google.com/drive/folders/1Y5N7oYjx6oMf9eZDHEewztqitbQaVyZW?usp = sharing.

All custom code developed for the analytical pipeline, including the multi-algorithm outlier detection consensus, multivariate characterization, and the web application implementation, is publicly available in the GitHub repository: https://github.com/jjhav88/Outlier-Detection-Framework.

### 2.4. Generative AI Statement

No generative artificial intelligence tools were used in the study design, data collection, analyses, or interpretation of this work.

### 2.5. Outlier Detection Module

A set of complementary outlier detection methods was applied to maximize sensitivity and specificity, based on a multi-algorithm consensus approach followed by an adaptive decision rule tailored to the distribution of each dataset.

Before applying the outlier detection methods, univariate (Shapiro–Wilk) and multivariate (Anderson–Darling) normality was assessed. Given the frequent non-normality distribution observed in gene expression data, non-parametric methods were prioritized in the consensus rule.

Univariate methods (per gene/marker)

- Interquartile Range (IQR): An observation is an outlier if value<Q1−1.5∗IQR or value>Q3+1.5∗IQR. A robust, non-parametric method.
- Z-Score: An observation is an outlier if $\left|z-score\right|>3$. A parametric method that assumes normality.
- Median Absolute Deviation (AD): An observation is an outlier if $\frac{\left|value-median\right|}{MAD}>3$. A robust, non-parametric method.

Multivariate methods (per sample)

- Mahalanobis Distance: An observation is an outlier if the distance exceeds X^2^ (0.975, *p*) for *p* variables. Assumes multivariate normality.
- Local Outlier Factor (LOF): Detects local outliers based on neighborhood density. A non-parametric method ideal for irregular clusters.
- Isolation Forest: Isolates outliers using random decision trees. An efficient, non-parametric method.

Statistical Hypothesis tests

- Grubbs’ Test: For detecting a single outlier in normally distributed data.
- Dixon’s Q-test: For small sample sizes (3–30).
- Generalized Extreme Studentized Deviate (ESD) Test (Rosner’s Test): For iteratively detecting multiple outliers in samples larger than 25.

### 2.6. Adaptive Consensus Strategy

To avoid duplicates and false positives, a voting system was implemented that adapts to data normality. Given the global non-normality, a data point was classified as a final outlier if it met one of the following criteria:

1. It was detected by any statistical hypothesis test (high confidence).
2. It was detected by at least two univariate non-parametric methods (IQR and/or MAD).
3. It was detected by at least one multivariate non-parametric method (LOF or Isolation Forest).

Parametric methods (Z-score and Mahalanobis Distance) were only considered if an individual variable passed the normality test.

### 2.7. Outlier Analysis Module

Samples or genes identified as final outliers were retained for an in-depth, multi-layered analysis:

- Comparative descriptive analysis: To compare clinical–demographic characteristics between outlier groups and normal data groups using the Mann–Whitney U test (with continuity correction and Benjamini–Hochberg FDR adjustment for multiple comparisons) for continuous variables and the chi-square or Fisher’s exact test (categorical variables).
- Advanced characterization analysis: Included a robust Principal Component Analysis (robust PCA), where the pcaGrid function from the rrcov package in R was applied. This validated, robust method detects atypical samples in RNA-seq data with greater precision than classical PCA. An unsupervised clustering approach, using algorithms such as K-means and agglomerative hierarchical clustering, was employed to identify subgroups within the outlier population.
- Co-expression network analysis: To understand the clinical and biological implications of the findings, a weighted gene co-expression network analysis was performed separately for the outlier and normal groups to identify gene modules associated with the atypical phenotype.
- Predictive modeling: A Random Forest model was trained to predict the outlier status based on clinical and gene expression features, thereby identifying the most important predictors of atypical status.

All statistical analyses were performed with a significance threshold of *p* < 0.05 and a 95% confidence interval (CI).

## 3. Results

### 3.1. Multi-Algorithm Consensus Framework for Evaluating Extreme Profiles in Diabetic Neuropathy

The cross-sectional study included a cohort of 93 adult patients with an established clinical diagnosis of diabetic neuropathy. Participants were balanced across two groups based on the intervention received: an electroacupuncture treatment group (n = 48) and a control group without this intervention (n = 45). The design included three temporal assessment points, with a constant sample size at each time point (n = 31): baseline (pre-intervention), second measurement (ME02), and third measurement (ME03).

Table 1 shows the distribution of participants according to the main categorical variables: intervention group (electroacupuncture vs. control) and assessment time (basal, ME02, ME03).

This distribution reflects a perfectly balanced design, with equal numbers of participants in each combination of group and time point. This balance ensures comparability between groups and overtime, minimizing potential biases arising from allocation or follow-up imbalances.

Given that the primary objective of the present study is to identify baseline extreme profiles independent of the intervention, and that the cohort exhibits a balanced distribution across groups and time points, the entire cohort is analyzed as a single dataset for outlier detection. This approach enables the capture of intrinsic extreme phenotypes within the pathophysiology of diabetic neuropathy, aligning with the proposed methodological strategy.

### 3.2. Identification and Detection of Outliers

To overcome the inherent limitations of univariate methods and their dependence on distributional assumptions, a rigorous outlier detection strategy based on an adaptive multi-algorithm consensus was implemented. Prior to detection, the normality of the dataset, comprising the expression profiles of eleven immunological mediators in 93 patients, was assessed. Univariate (Shapiro–Wilk) and multivariate (Anderson–Darling) normality analyses confirmed a globally non-normal distribution, with less than 5% of the variables meeting Gaussian assumptions. This classification activated the consensus rule, which prioritizes robust, nonparametric methods designed to maximize specificity and minimize false positives in complex biomedical data.

The applied battery of methods included: univariate non-parametric methods (Interquartile Range (IQR) and Median Absolute Deviation (MAD)); a univariate parametric method (Z-score (applied only if an individual variable passed the normality test, which was infrequent)); multivariate methods (Mahalanobis Distance (parametric), Local Outlier Factor (LOF), and Isolation Forest (non-parametric)); and statistical hypothesis tests (Grubbs, Dixon and Rosner).

**Figure 1:**
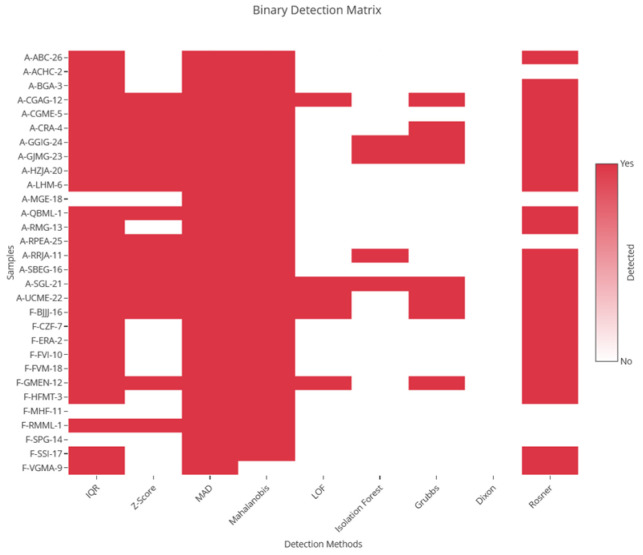
Multi-algorithm detection process and consensus convergence in the diabetic neuropathy cohort. A binary detection matrix (heatmap) showing the results of the 9 applied methods for the 93 samples (rows). Each red cell indicates the sample was identified by the corresponding method (column). A dispersed detection pattern is observed, with specific samples being flagged consistently by multiple algorithms.

The parallel application of these nine methods generated a broad initial spectrum of outlier candidates (Figure 1). To refine this set and establish a high-consensus and robust identification, the following adaptive decision rule was applied: an observation was classified as a final outlier if it met at least one of these three criteria: (1) being detected by any hypothesis test (Grubbs, Dixon, or Rosner); (2) being identified by at least two univariate non-parametric methods (IQR and MAD); (3) being flagged by at least one multivariate non-parametric method (LOF or Isolation Forest).

This filtering process converged on the robust identification of 44 observations as high-consensus outliers out of 93 total patients. This represents a prevalence of 47.3% in this diabetic neuropathy cohort. Redundancy in detection was notable: individuals such as A-GGIG-24, A-RRJA-11, A-GJMG-23, and A-SGL-21 were identified independently by five or more methods, validating the statistical robustness of these extreme observations.

### 3.3. Descriptive Analysis According to Outlier Status

Following the identification of 44 observations (47.3% of the cohort) as outliers, this group was descriptively characterized relative to the 49 individuals (52.7%) classified as normal. This initial analysis focused on the study design categorical variables and the expression profiles of the 11 immunological mediators assessed.

#### 3.3.1. Distribution of Categorical Variables

The distribution of outliers and normal data according to the categorical variables “observation group” and “assessment time point” was notably balanced (Table 2). No substantial deviations were observed in the proportion of participants undergoing electroacupuncture (54.2% in outliers vs. 42.9% in normal) or in the control group (45.8% vs. 57.1%). Similarly, the distribution across the three measurement time periods (Basal, ME02, ME03) was identical between the two groups, with exactly one-third of individuals in each category in both datasets. This homogeneity in categorical distribution reinforces the notion that the outlier phenotype is not artificially biased by treatment allocation or assessment timing, validating the approach of analyzing the cohort.

#### 3.3.2. Descriptive Profile of Immunological Mediator Expression

The descriptive analysis revealed drastic differences in gene expression patterns between the groups. As summarized in Table 3, for all evaluated markers, the outlier group exhibited consistently higher means and medians compared to the normal group, alongside a considerably greater dispersion (standard deviation). This pattern suggests not only an upward shift in central tendency but also extreme heterogeneity and the presence of exceptionally high individual values within the outlier group.

**Figure 2:**
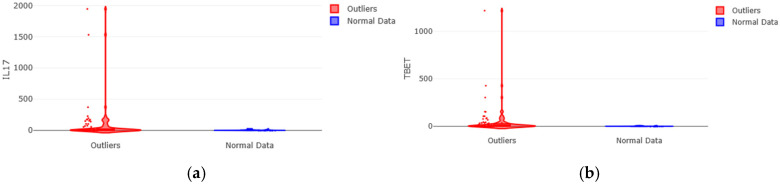
Most extreme gene expression profiles in the outlier subgroup of diabetic neuropathy. (**a**) IL-17. (**b**) T-bet. For each marker, a violin plot is shown with internal box plots and overlaid points representing individual observations. The distribution of the outlier group (red) exhibits extreme asymmetry, with a highly elongated right tail denoting the presence of exceptionally high values, in stark contrast to the compact distribution of the normal group (blue). Differences in the *Y*-axis scale (implicitly logarithmic due to the dispersion) are notable.

Two markers stood out for the magnitude of the intergroup discrepancy, representing the most extreme profiles. IL-17, the effector cytokine of the Th17 pathway, revealed a profound disparity: the mean in the outlier group was 20.6 times higher than that in the normal group. More relevantly, its maximum expression value exceeded the maximum observed in the normal group by a factor of 76.6 (1944.06 vs. 25.37) (Figure 2a). A similar pattern was observed for T-bet, the master transcription factor of the Th1 pathway, whose mean value in outliers was 33.8 times greater and whose maximum value was 153.3 times higher (1216.89 vs. 7.94) than those in the normal group (Figure 2b).

This pattern, in which medians are moderately higher but means and, crucially, maximum values are orders of magnitude greater in the outlier group, indicates a strongly right-skewed distribution. This suggests the existence of a subset of individuals within the outliers who exhibit an extremely elevated and disproportionate immune activation of the Th1 and Th17 pathways.

Collectively, this initial descriptive analysis reveals that the outlier phenotype in diabetic neuropathy is associated with a markedly altered immunological profile, characterized by a massive and heterogeneous elevation of key mediators in the Th1 and Th17 pathways, particularly IL-17 and T-bet. This establishes the foundation for subsequent inferential and multivariate analyses to confirm the statistical significance and biological relevance of these differences.

#### 3.3.3. Biochemical–Metabolic Profile in the Outlier Condition

Complementing the immunological characterization, the biochemical–metabolic profile of the observations classified as outliers was assessed. Descriptive analysis of variables such as uric acid, blood urea nitrogen (BUN), total cholesterol, creatinine, glucose, glycated hemoglobin (HbA1c), triglyceride (TG), and urea revealed an interesting pattern: while most parameters (uric acid, BUN, creatinine, glucose, HbA1c, urea) showed very similar distributions between outlier and normal groups, two variables stood out due to substantial differences.

Total cholesterol levels and, more pronouncedly, triglyceride levels were notably higher in the outlier group (Table A1). The median TG in outliers was 1.6 times greater than that in normal data (159.5 vs. 146.6 mg/dL). Still, the dispersion was extraordinary: the maximum value in the outlier group (834.3 mg/dL) exceeded the maximum in the normal group (327.3 mg/dL) by a factor of 2.5. This pattern of extreme elevation and high variability in TG replicates the distribution observed for key immunological markers (IL-17 and T-bet), suggesting a potential pathophysiological interconnection.

This convergence between a hyperactive immune profile (Th1/Th17) and marked dyslipidemia, particularly extreme hypertriglyceridemia, points towards an integrated immune–metabolic phenotype. The co-occurrence of these alterations in the outlier subgroup suggests that lipid dysregulation may constitute an integral component of the systemic pro-inflammatory state characterizing these patients. This finding provides a basis for subsequent multivariate analyses (network analysis and predictive modeling) to explore causal relationships and the interdependence between disproportionate activation and altered lipid metabolism in diabetic neuropathy with an extreme phenotype.

### 3.4. Analysis of Association Between Outlier Status and Categorical Variables

To determine whether the outlier status identified via the multi-algorithm consensus was significantly associated with the study’s categorical variables, chi-square tests of independence were performed. This analysis was crucial to rule out the possibility that the observed atypically was a byproduct of treatment allocation or assessment timing.

The chi-square test revealed no statistically significant association between outlier status and the intervention group variable (electroacupuncture vs. control) (χ^2^ = 0.441; *p* = 0.507; Table 4). The effect size, assessed by the Phi coefficient (φ = 0.069), was classified as small, confirming the absence of a clinically relevant relationship. Similarly, outlier status showed no association with the assessment point variable (basal, ME02, ME03) (χ^2^ = 0.000; *p* = 1.000), with an extremely small Cramer’s V effect size (0.0000).

These findings are methodologically robust, as all expected frequencies in the contingency tables are greater than or equal to 5, fulfilling the test’s assumptions. The complete absence of association with the assessment time point is particularly notable, given that the distribution of outliers is identical (14 observations, 33.3%) at each of the three measurement points.

Collectively, these results suggest that the outlier phenotype is not a consequence of the electroacupuncture intervention applied in this study, nor is it confined to a specific follow-up phase. Instead, these data support the hypothesis that outlier status represents an intrinsic, basal characteristic of a subset of patients with diabetic neuropathy. This characteristic may be rooted in pre-existing genetic, epigenetic, or environmental factors that predispose individuals to an extreme immune–metabolic state, independent of the assigned therapy or the time of observation. This finding validates the adopted analytical approach of treating the cohort as a single set for phenotype characterization, focusing on the underlying biology rather than the experimental design variables.

### 3.5. Comparison of Immunological and Metabolic Profiles Between Outlier and Normal Groups

To determine whether the differences observed descriptively in the gene expression and biochemical profiles between the outlier and normal groups reached statistical significance, the Mann–Whitney U test, appropriate for non-normal data, was applied to each immunological marker. To account for multiple comparisons, *p*-values obtained across all markers were adjusted using the Benjamini–Hochberg false discovery rate (FDR) procedure. The results, summarized in Table 5, reveal a pattern of specific and significant immune dysregulation in the outlier phenotype.

#### 3.5.1. Significant Differences in Immune Response Mediators

**Figure 3:**
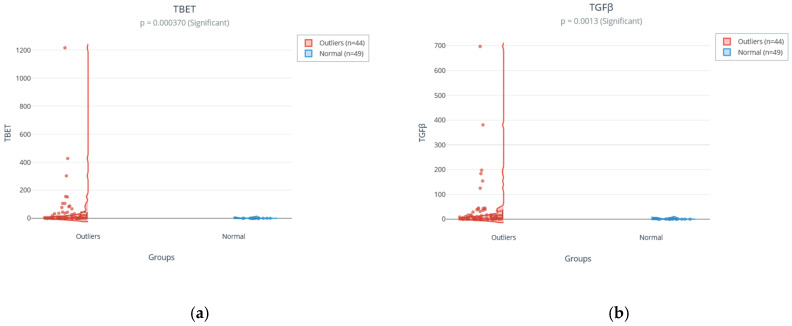
Significant differences in the most relevant immunological markers. (**a**) T-bet and (**b**) TGFβ, the two markers with the most robust differences (lowest *p*-value and largest effect size). Violin plots with internal box plots show the significantly higher distribution of these markers in the outlier group (red) compared to the normal group (blue), confirming the selective hyperactivation of these specific pathways.

Six of the eleven assessed immunological markers showed statistically significant differences (*p* < 0.05) between groups, following correction for multiple comparisons. Effect sizes were calculated using Rosenthal’s r to quantify the magnitude of the observed differences beyond statistical significance. T-bet, the master transcription factor of the Th1 pathway, emerged as the most robustly differentiated marker (Figure 3a), with the highest significance (*p* = 0.00037) and the largest effect size (r = 0.37), classified as medium as for the Rosenthal r coefficient. This indicates a pronounced hyperactivation of the Th1 pathway in outlier individuals.

Similarly, TGFβ (*p* = 0.0013; r = 0.33), SOCS3 (*p* = 0.0068; r = 0.28), STAT5 (*p* = 0.019; r = 0.24), SOCS1 (*p* = 0.018; r = 0.25), and STAT3 (*p* = 0.024; r = 0.24) showed significant differences with effect sizes ranging from small to medium (Figure 3b). These findings point to coordinated dysregulation involving not only effector cytokines but also key regulators of intracellular signaling (STATs) and negative feedback mechanisms (SOCS).

#### 3.5.2. Absence of Differences in Key Systemic Inflammation Markers

Notably, classic markers of systemic inflammation did not show significant differences. IL-17 (*p* = 0.17) and RORγT (*p* = 0.32), despite their descriptively extreme elevations, did not reach statistical significance in the inferential analysis. These results suggest that although IL-17 expression appeared elevated descriptively, the available data do not support a statistically significant difference between groups. More revealingly, TNF-α, a central mediator of inflammation in diabetes, did not differ between groups (*p* = 0.56), with a minimal effect size (r = 0.06).

The combination of these results suggests a canalized and specific immunological phenotype in outlier individuals. The pattern does not represent generalized inflammation (as indicated by an elevation in TNF-α), but rather a selective dysregulation of intracellular regulatory pathways (STAT3, STAT5, SOCS1, SOCS3) and the TGFβ/T-bet axis. This latter finding is particularly relevant, as T-bet is crucial for Th1 lymphocyte differentiation, and TGFβ possesses dual roles in immune regulation and fibrosis.

This profile of selective dysregulation, particularly the elevation of TGFβ and T-bet alongside the lack of change in TNF-α, could indicate a pro-fibrotic and immunomodulatory state rather than a purely pro-inflammatory one, with potential implications for the progression of diabetic neuropathy and the response to treatment.

### 3.6. Principal Component Analysis (PCA): Reconfiguration of the Immunological Space in the Outlier Phenotype

To investigate whether individuals identified as outliers differ not only in the magnitude of individual markers but also in the multivariate structure of their global immunological profile, a robust Principal Component Analysis (robust PCA) was performed. This analysis was applied to the standardized matrix of the 11 measured immunological mediators (IL-17, RORγT, SOCS1, SOCS3, STAT3, STAT4, STAT5, STAT6, T-bet, TGFβ, TNF-α) across all 93 observations.

#### 3.6.1. Component Selection and Variance Structure

**Figure 4:**
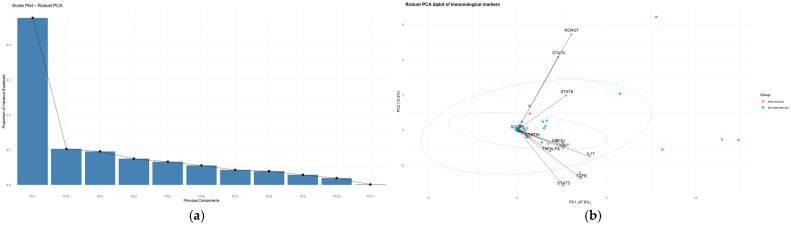
Robust Principal Component Analysis (robust PCA) of the immunological profile. (**a**) Scree plot showing the proportion of variance explained by each principal component. (**b**) Robust PCA biplot (PC1 vs. PC2). Points represent individual observations (blue: normal group, n = 49; red: outlier group, n = 44). Arrows (vectors) indicate the direction and magnitude of the loadings of the immunological markers. Outlier samples occupy more peripheral regions of the multivariate space, evidencing distinct combinations of immune signaling alterations.

Inspection of the scree plot (Figure 4a) and the proportion of explained variance guided the interpretation of the principal components (PCs). The first component (PC1) explained 47.6% of the total variance, followed by PC2 (10.2%), PC3 (9.5%), PC4 (7.5%), and PC5 (6.6%). Together, the first five components accounted for approximately 81.4% of the total variance (Table 6).

The loading structure revealed that PC1 was positively influenced by multiple markers, including IL-17, TGFβ, STAT3, T-bet, SOCS1, and TNF-α, indicating that this component reflects a broad axis of coordinated immune activation involving several inflammatory and regulatory pathways.

PC2 displayed a contrasting structure, with strong positive contributions from RORγT, STAT4, and STAT6, while IL-17 and TGFβ contributed negatively. This pattern suggests that PC2 captures a dimension reflecting variation in Th17-related signaling and downstream regulatory pathways.

#### 3.6.2. Spatial Segregation of the Outlier Phenotype in the Multivariate Space

Projection of observations onto the plane defined by the first two principal components (PC1 and PC2) reveals a clear spatial segregation between outlier and non-outlier individuals (Figure 4b). While observations from the normal group form a compact cluster near the center of the PCA space, outlier samples occupy more peripheral and dispersed positions.

This distribution indicates that the outlier phenotype is not driven by the extreme value of a single biomarker but rather by distinct multivariate combinations of immunological dysregulations. Outliers are distributed along both principal axes, suggesting heterogeneity in their atypical profiles: some individuals exhibit high scores along the integrated activation axis (PC1), whereas others show divergence along the secondary axis captured by PC2.

The robust PCA therefore supports the interpretation that the detected outliers represent transcriptionally distinct immunological states characterized by coordinated alterations across multiple signaling pathways. The clear separation observed in the first two components (explaining 57.8% of the variance) illustrates how multivariate approaches can reveal complex biological structures that remain undetectable using univariate analyses.

### 3.7. Co-Expression Network Architecture in the Outlier Phenotype

To investigate whether outlier status is associated with fundamental alterations in the functional relationship between genes and clinical parameters, beyond changes in individual magnitude, a comparative co-expression network analysis was performed. Independent networks based on Spearman correlations were constructed for the outlier (n = 44) and normal (n = 49) groups, integrating the 11 immunological variables (IL-17, RORγT, SOCS1, SOCS3, STAT3, STAT4, STAT5, STAT6, T-bet, TGFβ, TNF-α) and 8 biochemical–metabolic variables (HbA1c, glucose, cholesterol, TG, creatinine, BUN, uric acid, urea). Significance (*p* < 0.05) and correlation strength (|r| > 0.5) thresholds were applied consistently.

#### General Topology and Network Density

The outlier group network exhibited a greater density of significant connections, with 37 edges (connections) among the 19 nodes, compared to the 30 edges observed in the normal group network. This 23% increase in connectivity suggests a state of tighter coupling or coordination between immunological and metabolic pathways in the extreme phenotype, possibly reflecting a more integrated systemic dysregulation.

Comparative analysis of node centrality (measured by normalized degree) revealed a substantial topological reorganization in the outlier group (Table 7). Multiple intracellular signaling regulators and cytokines gained prominence as major hubs unique to the outlier network.

The most dramatic changes were observed in TGFβ, which transitioned from a peripheral node in the normal network (centrality = 0.056) to one of the most central hubs in the outlier network (centrality = 0.444; Δ = +0.389). Similarly, STAT4 substantially increased its centrality (Δ = +1.278). In stark contrast, metabolic variables such as uric acid and glucose, which functioned as moderate hubs in the normal network, lost all their significant connections in the outlier network (Δ = −0.222 each).

**Figure 5:**
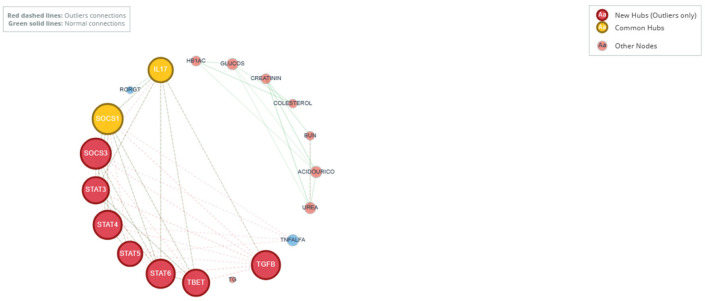
Comparative co-expression network analysis. Schematic representations of the networks derived for the normal group (solid gray connections) and outlier group (dashed red connections) are overlaid. Nodes (circles) represent variables; their size is proportional to degree centrality. Key topological changes are highlighted: in the outlier network, TGFβ and STAT4 emerge as new high-centrality hubs (large nodes with thick borders), while uric acid and glucose have drastically reduced size and connectivity. SOCS1 and IL-17 (yellow) are common on both networks.

The overlay of both networks in an integrated network graph (Figure 5) allows visualizations of this fundamental topological shift. Connections conserved in the normal network are shown as solid gray lines, while new connections specific to the outlier network are represented as dashed red lines. Node size is proportional to its centrality degree within its respective network.

This finding is consistent with the univariate analysis, which showed a significant elevation in TGFβ and T-bet, and reinforces the notion of a phenotype canalized toward specific pathways of tissue remodeling and immune regulation, rather than classic acute inflammation (TNF-α was not a hub in either network). The outlier network therefore reflects a distinct pattern of coordinated immune–metabolic interactions that may contribute to the biological heterogeneity observed in diabetic neuropathy.

### 3.8. Analysis of Unsupervised Clustering Algorithms

#### 3.8.1. Evaluation of Clustering Tendency in the Diabetes Neuropathy Cohort

Prior to applying clustering algorithms to identify sub-phenotypes within the outlier group, it was essential to determine whether the data exhibits an underlying structure that justifies the search for natural groupings. Applying clustering to data without inherent structure can generate spurious clusters. Therefore, a rigorous evaluation of clustering tendency was conducted using a multimodal approach that combined metrics and visual assessment.

The Hopkins statistic was calculated, a measure that compares the distribution of distances in the real dataset with that of a uniformly random dataset in the same dimensional space. A value of 0.5 indicates complete randomness, while values close to 1 suggest a strong clustering tendency. For the outlier subgroup dataset (n = 44 observations, 11 immunological variables), the Hopkins statistic was 0.8563. Although this value is above 0.5, it indicates a moderate clustering tendency rather than a strong one, suggesting that while some structure may exist, the data also contain considerable heterogeneity or noise.

**Figure 6:**
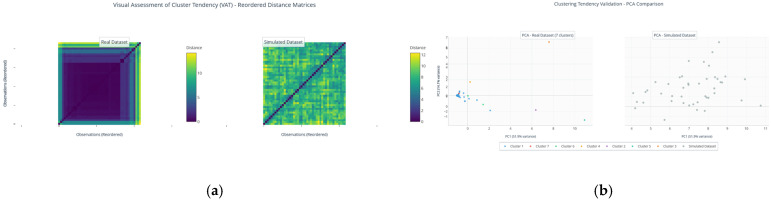
Evaluation of cluster tendency. (**a**) Reordered Euclidean distance matrices (VAT) for the real dataset (left) and a simulated random dataset (right). The presence of dark square blocks along the diagonal in the real dataset is a diagnostic pattern indicating the existence of natural clusters. (**b**) PCA projections (PC1 vs. PC2) of the real dataset (colored by preliminary automatic clusters) and the simulated data (gray). The real data show discernible groupings and structure, in contrast to the uniform distribution of the simulated data.

For complementary visual validation, reordered Euclidean distance matrices (Visual Assessment of Cluster Tendency, VAT) were generated for both the real dataset and the simulated random one (Figure 6a). The matrix of the real dataset showed the formation of clearly defined dark blocks along the diagonal, a diagnostic pattern indicating the existence of natural, compact clusters. In stark contrast, the matrix of the simulated dataset presented a homogeneous, random pattern with no discernible blocks.

Finally, the projection of both datasets onto the space of the first two principal components (PC1 and PC2) corroborated this difference (Figure 6b). While the simulated dataset was uniformly distributed and structureless, the real dataset showed an arrangement with discernible groupings and clearly separated peripheral observations, consistent with the presence of outliers and subpopulations.

Collectively, the Hopkins statistics and visual evaluations (VAT and PCA) robustly confirm the presence of a moderate inherent clustering structure within the outlier subgroup. This finding provides a cautious justification for the subsequent application of clustering algorithms to explore sub-phenotypes within this atypical population.

#### 3.8.2. Determination of the Optimal Number of Clusters (k) in the Outlier Group

With clustering tendency confirmed as moderate, the optimal number of clusters (k) for partitioning the data was determined. A range of k from 2 to 10 was evaluated using four widely recognized internal validation indices: the Elbow method (based on Within-Cluster Sum of Squares, WSS), the Silhouette Coefficient (Silhouette score), the Calinski–Harabasz index (CH), and the Davies–Bouldin index (DB).

The results revealed a divergence between methods, each highlighting different aspects of the data structure. The Elbow method suggested k = 4 as a point of diminishing returns in variance explained. The Silhouette Coefficient, considered one of the most robust methods for assessing cluster cohesion and separation, reached its maximum value at k = 2 (Silhouette score = 0.7740). In contrast, the CH index increased monotonically across the tested range, favoring the maximum evaluated solution (k = 10), while the DB index was minimized at k = 6.

Faced with this methodological divergence, we adopted a decision strategy that prioritizes interpretative robustness, biological parsimony, and the primary objective of identifying major discrete sub-phenotypes within the outliers. While indices like CH are sensitive to finding many small subgroups in high-dimensional data, they can overfit subtle variations. Therefore, k = 2 was selected as the optimal number of clusters for subsequent analysis. This decision is based on (1) the optimization of the Silhouette Coefficient, which directly measures how well each observation fits its assigned cluster; (2) the principle of parsimony favoring a simpler model that captures the most fundamental split in the data; (3) coherence with the overarching goal of defining broad, interpretable immune–metabolic subtypes among the extreme observations.

#### 3.8.3. K-Means and Agglomerative Hierarchical Clustering Algorithms

To unravel the underlying structural heterogeneity within the outlier diabetic neuropathy cohort (n = 44) and validate the existence of biologically relevant sub-phenotypes, a multimodal unsupervised clustering approach was applied. Two independent and complementary algorithms were employed: K-means (partitional) and agglomerative hierarchical clustering (with complete linkage and Euclidean distance). This dual strategy allowed for the assessment of clustering robustness, stability, and reproducibility, minimizing the risk of algorithmic artifacts.

Both clustering methods converged robustly on an identical bipartition of the outlier subgroup (Table 8). The K-means algorithm partitioned the 44 observations into two clusters: Cluster 0, comprising 3 individuals (6.8%), and Cluster 1, comprising the remaining 41 individuals (93.2%). Agglomerative hierarchical clustering, when cut at the corresponding height (k = 2), produced a perfectly congruent assignment, underscoring the robustness of this fundamental division within the outlier population.

**Figure 7:**
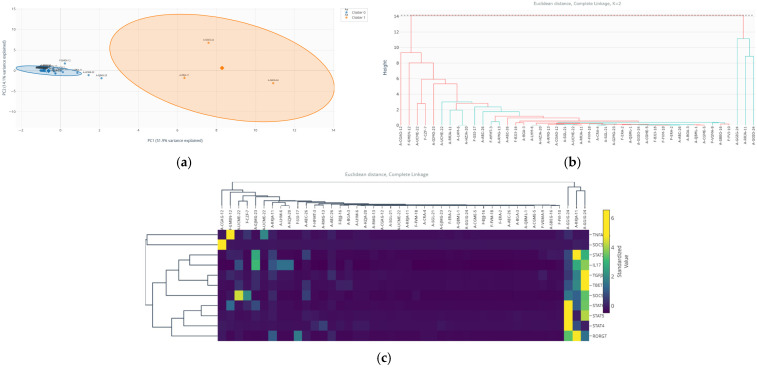
Identification and characterization of discrete immunological sub-phenotypes within the outlier subgroup via unsupervised clustering. (**a**) Projection onto the PCA plane (PC1 vs. PC2) of the hierarchical algorithm results (k = 2) applied to the 44 outlier observations. Points are colored by cluster assignment: orange (Cluster 0, n = 3, ultra-extreme phenotype) and purple (Cluster 1, n = 41, core outlier phenotype). Ellipses represent 95% confidence intervals. The clear spatial segregation highlights the distinct multivariate profile of Cluster 0. (**b**) Dendrogram of agglomerative hierarchical clustering (Euclidean distance, complete linkage). The early and deep split (dashed line) separates the branch containing the three individuals of Cluster 0 (orange) from the main branch comprising Cluster 1 (purple), independently validating the partition. (**c**) Heatmap showing the standardized expression profiles (Z-score) of the 11 immunological markers, ordered according to the dendrogram structure. The pattern of massive, coordinated hyper-expression across multiple markers (IL-17, T-bet, STAT4, SOCS3, TGFβ) in Cluster 0 (orange sidebar) contrasts with the more moderate and heterogeneous profile of Cluster 1 (purple sidebar).

The projection of these clusters onto the plane defined by the first two principal components (PC1 and PC2) reveals a striking and clear spatial segregation (Figure 7a). Cluster 0 occupies an extreme, peripherical position at the positive extreme of PC1, far removed from the central cloud formed by Cluster 1. This spatial isolation in the multivariate immunological space confirms that these three individuals represent a transcriptionally distinct and highly atypical sub-phenotype even within the already extreme outlier group.

The dendrogram generated by agglomerative hierarchical clustering (Figure 7b) exhibits an early and deep branching that unequivocally separates the three individuals forming Cluster 0 from the main branch containing all 41 individuals of Cluster 1. This hierarchical structure independently validates the partition and visually emphasizes the substantial dissimilarity of this small subgroup from the remaining outliers.

The heatmap coupled with the dendrogram (Figure 7c), showing standardized expression profiles (Z-scores) of the 11 immunological markers, enables characterization of distinct molecular patterns. Cluster 0 is characterized by a massive, coordinated hyper-expression across nearly all evaluated markers, particularly IL-17, T-bet, STAT4, SOCS3 and TGFβ. This profile suggests a state of global, dysregulated immune activation, with potent co-activation of the Th1, Th17, and JAK-STAT signaling pathways. In contrast, Cluster 1 shows a more moderate and heterogeneous elevation of the same markers, representing the core atypical phenotype: while deviant from the normal population, it is internally more consistent and less extreme than Cluster 0.

The robust convergence of two independent clustering algorithms unequivocally identifies a fundamental dichotomy within the outlier subgroup in diabetic neuropathy. Beyond a shared atypical status, there exists a rare, ultra-extreme immunologic sub-phenotype (Cluster 0, 6.8%) characterized by a massive hyper-inflammation, distinct from the more common outlier profile (Cluster 1, 93.2%). This finding powerfully reinforces the central thesis of our study: systematic outlier investigation not only separates atypical from normal individuals but can further stratify the outliers themselves, revealing additional layers of heterogeneity within the outlier population. The presence of this small cluster suggests the existence of highly atypical expression patterns that may reflect distinct biological processes, although further studies are required to determine their mechanistic or clinical relevance.

### 3.9. Predictive Analysis Using Random Forest: Identification of Key Determinants of the Outlier Phenotype

To identify the most relevant clinical, biochemical, and immunological variables for predicting the outlier phenotype and to quantify their discriminative capacity, a supervised Random Forest model was constructed. This algorithm, robust to nonlinear relationships and high dimensionality, enables evaluation of the relative importance of each predictor and estimation of the model’s overall accuracy in classifying individuals based on their atypicality status.

#### 3.9.1. Model Design and Training

A Random Forest classifier was trained using the binary outlier vs. normal condition, defined by the previously described multi-algorithm consensus, as the response variable. Nineteen variables integrating biochemical, metabolic, and immunological dimensions were included as predictors: eight biochemical variables (uric acid, BUN, total cholesterol, creatinine, glucose, HbA1c, triglycerides, urea) and eleven gene expression markers (IL-17, RORγT, SOCS1, SOCS3, STAT3, STAT4, STAT5, STAT6, T-bet, TGFβ, TNF-α). The data was split into training (n = 74, 79%) and testing (n = 19, 20.4%) sets while maintaining class proportions. The model was optimized with the following hyperparameters: 50 trees, maximum depth = 7, minimum samples to split a node = 3, and minimum samples per leaf = 1.

#### 3.9.2. Model Predictive Performance

The model demonstrated solid discriminative capability on the test set (Table 9). The area under the ROC curve (AUC-ROC) was 0.783, indicating a good ability to distinguish between outlier and normal individuals. Accuracy reached 84.2%, with a positive predictive value (precision) of 83.3% for the outlier class and a sensitivity (recall) of 100%, reflecting that the model correctly identified all true outliers in the test set.

**Figure 8:**
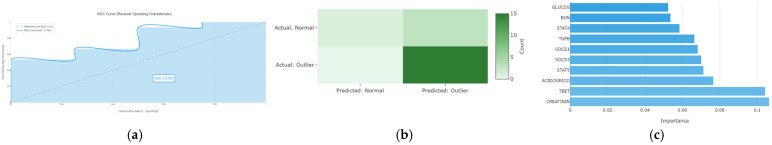
Predictive analysis of the outlier phenotype using Random Forest. (**a**) ROC curve of the model on the test set (AUC = 0.783). (**b**) Confusion matrix showing the distribution of correct and incorrect classifications for the normal and outlier classes. (**c**) Variable importance plot (top 10) based on the mean decrease in Gini impurity, reflecting each variable’s contribution to the model’s classification capability.

The ROC curve (Figure 8a) shows consistent performance above the reference line, supporting the model’s potential clinical utility. The confusion matrix (Figure 8b) details that, of the 19 individuals in the test set, 10 were correctly classified as normal, 5 were correctly classified as outliers, and there were 4 false positives (normal individuals classified as outliers).

The variable importance analysis (calculated as the mean decrease in Gini impurity) revealed a set of high-impact predictors, with contributions distributed between biochemical and immunological variables (Figure 8c, Table 10). The ten most important variables together accounted for over 70% of the model’s predictive capacity.

Two key findings can be highlighted: First, the results predict an integrated biochemical–immunological function, where variables of renal function (creatinine, uric acid, BUN) and metabolism (glucose) appear among the top predictors, alongside immunological markers (TGFβ) and key transcription factors for T-cell activation (T-bet, STAT5, SOCS1, SOCS3, STAT4). This reinforces the notion of an integrated immune–metabolic phenotype underlying the outlier profile. Second, the predominance of intracellular regulators is evident: among the immunological markers, it is not the classic effector cytokines (IL-17, TNF-α) that are most relevant, but rather regulators of intracellular signaling (STATs, SOCS) and the transcription factor T-bet. This is consistent with prior findings of specific dysregulation in Th1 and JAK-STAT signaling pathways.

The Random Forest model not only validates the capacity of the measured variables to predict the outlier phenotype with high accuracy but also hierarchizes their relative contributions. The prominence of T-bet and STAT5/STAT4 underscores the central role of Th1 pathway hyperactivation and cytokine signaling in defining the extreme profile. Simultaneously, the relevance of creatinine, uric acid, and BUN suggests a pathophysiological interconnection with renal function, possibly reflecting a state of systemic metabolic stress or a shared organ vulnerability.

The successful integration of biochemical and immunological predictors into a single model with an AUC > 0.78 supports the hypothesis that the outlier phenotype in diabetic neuropathy is a multimodal construct in which metabolic and immune activation are mutually reinforcing. This integrated biochemical–immunological profile may represent a candidate signature associated with the outlier phenotype, although its clinical predictive value requires validation in larger independent cohorts.

To evaluate the robustness of the detected outlier profiles, a bootstrap resampling analysis (1000 iterations) was performed by repeatedly resampling the dataset with replacement and reapplying the complete outlier detection pipeline. The analysis showed moderate stability in the identification of extreme observations (mean detection stability ≈ 0.40), with several samples consistently classified as outliers in more than 60% of bootstrap iterations. These findings indicate that the detected observations represent reproducible multivariate deviations rather than artifacts produced by sampling variability, supporting the stability of the consensus-based detection framework.

### 3.10. Multi-Algorithm Consensus Framework for Evaluating Extreme Profiles in a 3T3-L1 Adipocyte Inflammation Model

As a fundamental initial step to contextualize subsequent gene expression analyses and extreme value detection, the structure and distribution of the in vitro study’s experimental design were characterized. The experiment evaluated the effects of glycine on cytokine expression in 3T3-L1 adipocytes under various gene-silencing and treatment conditions. The data matrix was structured around two key categorical variables: “group” (defined by the silencing status of TNF-α receptor subtypes) and “treatment” (defined by the stimulus applied to the cells). The “group” comprised three conditions: basal (cells without silencing) and wild-type cells; siRNA Tnfrsf1a (cells with silencing of the TNF-α receptor type 1a gene); and siRNA Tnfrsf1b (cells with silencing of the TNF-α receptor type 1b gene). The “treatment” comprised five conditions: control (culture medium + 10% Fetal Bovine Serum (FBS)); glycine (culture medium + 10% FBS + treatment with 10 mM glycine); TNF-α (culture medium + 10% FBS + treatment with TNF-α (5 ng/mL)); TNF-α + glycine (co-treatment with TNF-α (5 ng/mL) and 10 mM glycine); and glycine + TNF-α (culture medium + 10% FBS + pre-treatment with glycine followed by TNF-α (pre-incubation condition)).

**Figure 9:**
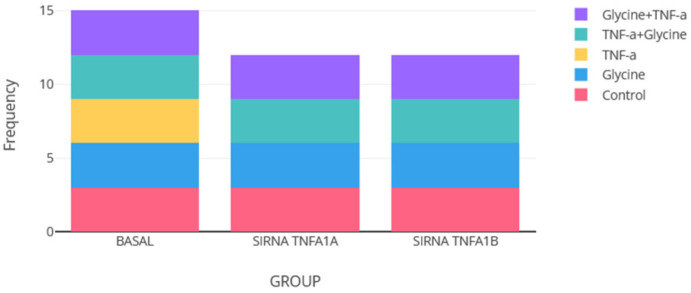
Distribution of the experimental design in the in vitro study with 3T3-L1 adipocytes. Stacked bar chart showing the distribution of the 39 experimental samples according to the categorical variables “group” (bar color) and “treatment” (subdivision within each bar). Each bar represents the total sample count for a group. The absence of the TNF-α condition in the silencing groups is evident.

The distribution of biological replicates across these conditions is shown in Figure 9 The design was balanced for the control, glycine, TNF-α + glycine, and glycine + TNF-α conditions, with three independent replicates (n = 3) per “treatment” condition within each “group”. A critical exception to this balance was the complete absence of replicates in the TNF-α only treatment condition within the silencing groups (siRNA Tnfrsf1a and siRNA Tnfrsf1b). This resulted in a total of n = 39 independent samples (cultures) available for subsequent gene expression analysis: 15 from the basal subgroup (5 treatments × 3 replicates) and 12 per silencing group (4 treatments × 3 replicates, excluding TNF-α only).

This initial characterization confirms that the in vitro dataset provides a robust platform for evaluating the specific effects of glycine and Tnfrsf1a/b silencing on cytokine expression. The design structure, although not fully balanced due to the intentional exclusion of certain conditions, is logically aligned with the study’s hypothesis and allows for statistically meaningful comparisons within well-defined experimental blocks. The next phase of the analysis applied the outlier detection strategy to the gene expression matrix derived from these 39 observations.

#### 3.10.1. Identified Outliers

To unravel extreme gene expression observations within the in vitro model, the adaptive consensus-based outlier detection strategy was applied to the gene expression matrix comprising the 39 samples. Formal assessment of univariate and multivariate normality (Shapiro–Wilk and Anderson–Darling) confirmed that the dataset was globally non-normal, with only 2% of the variables following a Gaussian distribution. This classification activated prioritization or robust, nonparametric methods within the consensus logic.

**Figure 10:**
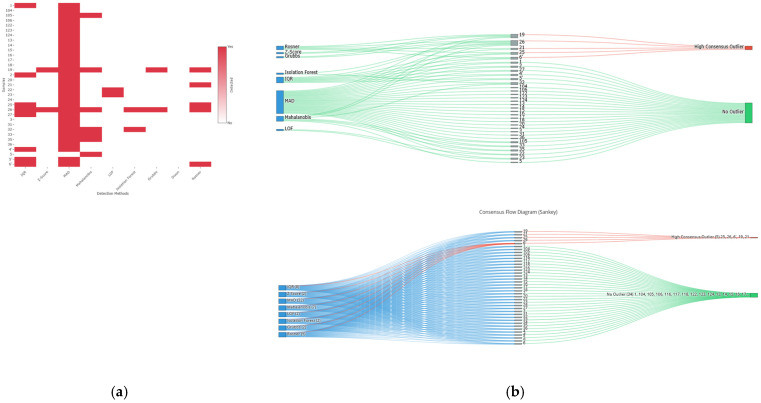
Multi-algorithm detection process and consensus convergence in the in vitro model. (**a**) Binary detection matrix (heatmap) showing the results of the 9 methods applied to the 39 samples (rows). Each red cell indicates that the sample (ID) was identified by the corresponding method (column). A scattered detection pattern is observed, with some samples (6, 27) being flagged by multiple methods. (**b**) Sankey flow diagram illustrating the adaptive consensus process. The nodes on the left represent each detection method. The flows (connections) show the samples identified by each method converging towards central nodes representing inclusion criteria. Only the flows that meet the consensus criteria (samples 6, 19, 21, 25, 26) converge at the final “high consensus” node, while unconfirmed detections are filtered out.

The parallel application of the battery of six detection algorithms and three statistical hypothesis tests generated a broad spectrum of initial candidates, as shown in the binary detection matrix (Figure 10a). This heatmap reveals a scattered pattern of detections, highlighting samples such as IDs 26 and 27, which were flagged by multiple methods. The consensus strategy, designed to maximize specificity by requiring multiple independent confirmations, acted as a rigorous filter. This process is illustrated in the Sankey flow diagram (Figure 10b).

The application of this decision rule converged on the robust identification of five observations as high-consensus outliers (sample IDs: 6, 19, 21, 25, 26), representing a prevalence of 12.8% (5/39) in this experimental dataset. Redundancy in detection validates the robustness of these atypical observations: sample ID 26 was independently identified by the Z-score, MAD, Mahalanobis, Isolation Forest, and Grubbs’ test methods.

#### 3.10.2. Descriptive Analysis of Gene Expression Profiles in Outliers from the In Vitro Model

Following the application of the multi-algorithm consensus framework for outlier detection, five samples (12.8% of the total) were identified as high-consensus observations (ID: 6, 19, 21, 25, 26). A comparative analysis of categorical and quantitative variables between the outlier (n = 5) and normal (n = 34) groups is presented next, with the aim of characterizing the biological profile associated with these extreme observations.

Table 11 summarizes the distribution of the identified outliers across the two main categorical variables of the experimental design: the “group” (silencing status) and the “treatment” applied.

As for the “group”, most outliers (60%) were found in the basal condition (no silencing), followed by the group with Tnfrsf1b silencing (40%). No outliers were identified in the group with Tnfrsf1a silencing.

As for the “treatment”, outliers were concentrated in the TNF-α (40%) and control (40%) conditions, with a single observation in the TNF-α + glycine condition (20%). No outliers were detected in the conditions involving glycine treatment alone or glycine pre-treatment.

**Figure 11:**
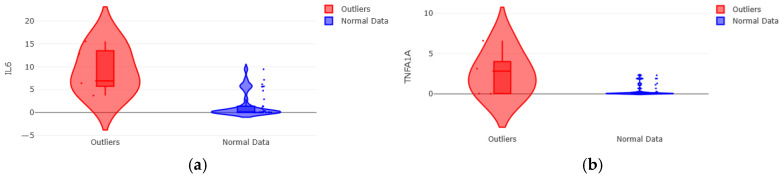
Extreme gene expression profile in outliers from the 3T3-L1 in vitro model. (**a**) Distribution of IL-6 (violin plot with overlaid points). Outliers (red) show notably elevated levels compared to the normal group (blue). (**b**) Distribution of the Tnfrsf1a. Expression of the type 1a receptor is markedly increased in the outlier group. Plots include the median and IQR. All measures are in normalized relative expression units.

The cytokine IL-6 showed the most pronounced alteration (Figure 11a). The median expression was 254 times higher in outliers (6.92; IQR: 3.71–15.56) than in the normal group (0.03; IQR: 0.00–0.58). Similarly, the receptor Tnfrssf1a exhibited notably elevated expression in outliers (Figure 11b), with a median 39 times higher (2.82 (IQR: 0.03–6.60) vs. 0.07 (IQR: 0.02–0.32) in the normal group). In contrast, the anti-inflammatory cytokine IL-10 showed a drastic reduction in outliers, with a median close to zero (0.003 vs. 1.47 in the normal group). The receptor Tnfrsf1b showed a trend towards lower expression in outliers (median: 0.32 vs. 0.31), although with overlapping ranges.

### 3.11. Association of Outliers with Experimental Variables and Comparison of Gene Expression Profiles

To determine whether outlier status was significantly associated with the experimental conditions and to characterize their expression profiles statistically, univariate inferential analyses were conducted.

#### 3.11.1. Association with Categorical Variables

The independence between outlier status (yes/no) and the two categorical design variables was assessed using the chi-square test. Given the small sample size and the presence of expected frequencies below 5, a Monte Carlo simulation was employed to ensure robustness.

The analyses revealed that the distribution of outliers was not statistically significantly associated with either the “group” (silencing condition; χ^2^ = 2.62; *p* = 0.796; Cramer’s V = 0.26) or the “applied treatment” (χ^2^ = 11.16; *p* = 0.152; Cramer’s V = 0.54) (Table 12). Although the test for “treatment” did not reach conventional significance, the effect size was large (Cramer’s V = 0.54), reflecting the observed distribution where outliers were concentrated in the TNF-α and control conditions.

#### 3.11.2. Comparison of Gene Expression Profiles Between Groups

To assess differences in the expression levels of the evaluated markers between the outlier (n = 5) and normal (n = 34) groups, the Mann–Whitney U test was applied. The results, including the U statistics, *p*-value, and effect size (Rosenthal’s r), are summarized in Table 13.

Among all evaluated markers, only IL-6 showed a statistically significant difference with a medium-to-large effect size between the groups (U = 159.0; *p* = 0.002; r = 0.50). The median IL-6 expression was 200 times higher in the outlier group.

Although Tnfrsf1a showed a marked descriptive elevation in outliers (median 39 times greater), this difference did not reach statistical significance in this reduced cohort (*p* = 0.186). Similarly, the drastic reduction in IL-10 and the downward trend in Tnfrsf1b in the outlier group approached the threshold for significance (*p* = 0.097 and *p* = 0.239, respectively), with effect sizes in the small-to-medium range.

The markers TNF-α and ADIPOQ showed no statistically significant differences or meaningful effect sizes between the groups.

Overall, the inferential analysis confirmed that the extreme expression profile of the outlier is primarily defined by a significant overproduction of IL-6, supporting the initial descriptive observation. The associations with the experimental conditions, while not statistically significant at this sample size, showed distribution patterns and effect sizes that warrant investigation in studies with greater power.

### 3.12. Multivariate Analysis of Expression Profiles: Reconfiguration of the Transcriptional Space in In Vitro Outliers

To determine whether outliers in the 3T3-L1 adipocyte model not only differ in the magnitude of individual markers but also occupy unique positions within the multivariate architecture of gene expression, a robust Principal Component Analysis (robust PCA) was performed.

#### Component Selection and Variance Structure

**Figure 12:**
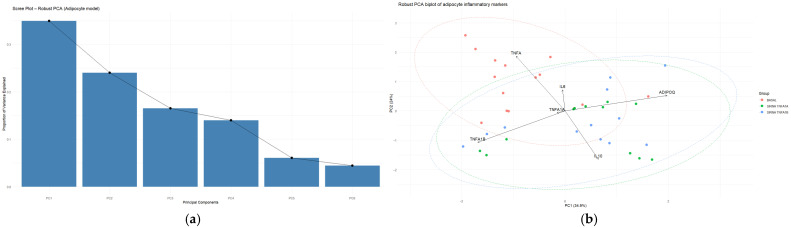
Robust Principal Component Analysis of the gene expression profile in 3T3-L1 adipocytes. (**a**) Scree plot showing the proportion of variance explained by each principal component. (**b**) Robust PCA biplot (PC1 vs. PC2). Points represent individual samples grouped by experimental conditions. Arrows (vectors) indicate the direction and magnitude of the loadings for the six analyzed markers (IL-10, ADIPOQ, IL-6, TNF-α, Tnfrsf1a, Tnfrsf1b). Samples positioned toward peripheral regions of the multivariate space represent atypical transcriptional profiles relative to the central cluster of observations.

Robust PCA was applied to the standardized expression matrix of six key markers (IL-10, ADIPOQ, IL-6, TNF-α, Tnfrsf1a, Tnfrsf1b) across the 39 experimental samples. Analysis of the scree plot and the proportion of explained variance guided the interpretation of the principal components (PCs) (Figure 12a). The first component (PC1) explained 34.9% of the total variance, followed by PC2 (24.0%) and PC3 (16.5%). Together, the first three components accounted for approximately 75.5% of the total variance (Table 14).

First, PC1 (34.9% of the variance) suggest an axis reflecting the balance between adipogenic/anti-inflammatory signaling and TNF-receptor-associated inflammatory pathways. This component was positively influenced by ADIPOQ and, to a lesser extent, Tnfrsf1a, whereas Tnfrsf1b contributed to the opposite direction. Therefore, PC1 represents a gradient ranging from a metabolically favorable and anti-inflammatory state (characterized by higher adiponectin expression) to a state more strongly associated with TNF-receptor-mediated inflammatory signaling.

Second, PC2 (24.0% of the variance) suggests an axis associated with variation in inflammatory cytokine activity. This component showed positive contributions from TNF-α and IL-6, while IL-10 contributed negatively. This pattern suggests that PC2 captures a gradient between pro-inflammatory activation (higher TNF-α and IL-6 expression) and anti-inflammatory regulatory responses mediated by IL-10.

Finally, PC3 (16.5% of the variance) reflects additional variation related to coordinated metabolic and inflammatory regulation within the adipocyte model. This component was influenced by contributions from IL-10 and ADIPOQ, indicating that part of the transcriptional variability may involve coordinated modulation of anti-inflammatory signaling and adipocyte functional markers.

The projection of samples onto the plane defined by PC1 and PC2 reveals a structured distribution of the experimental observations (Figure 12b). Samples from the different experimental conditions occupy partially distinct regions of the multivariate space, with several observations positioned toward the periphery of the distribution, indicating atypical transcriptional configurations.

These peripheral observations correspond to samples exhibiting extreme combinations of inflammatory and metabolic gene expression patterns relative to the central cluster formed by most of the observations.

On PC2, these atypical observations also exhibit disperse positions, reflecting heterogeneity in the balance between pro-inflammatory cytokine signaling (TNF-α and IL-6) and anti-inflammatory regulation mediated by IL-10.

This distribution confirms that the outlier phenotype is not the result of variation in a single gene but rather emerges from distinct multivariate combinations of inflammatory and metabolic expressions patterns, characteristics that becomes evident only through multivariate analyses integrating both pro- and anti-inflammatory markers.

### 3.13. Gene Co-Expression Architecture in the In Vitro Outlier State

To investigate whether outlier status was associated not only with changes in expression magnitude but also with fundamental alterations in the functional relationships between genes, a comparative co-expression network analysis was performed.

#### 3.13.1. General Network Topology

Co-expression networks based on Spearman correlations were constructed independently for the outlier (n = 5) and normal (n = 34) groups, using the same set of six markers. Significance (*p* < 0.05) and correlation strength (|r| > 0.8) thresholds were applied consistently to ensure comparability.

The topology of both networks was notably sparse. The normal group network presented two significant connections between marker pairs, while the outlier group network, despite its small sample size, also maintained two significant connections, suggesting partial preservation of some core relationships.

#### 3.13.2. Changes in Hub Identity and Centrality

**Figure 13:**
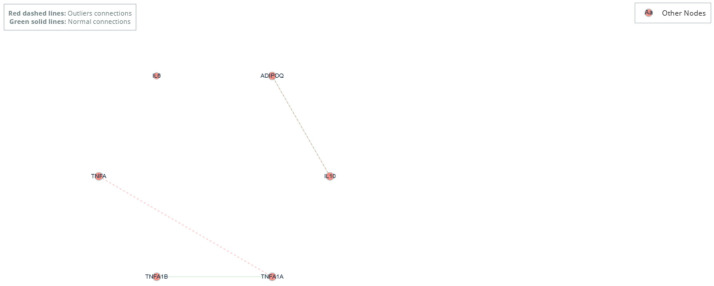
Gene co-expression network analysis in the 3T3-L1 model. Integrated comparative network. Both networks are overlaid. Connections from the normal network are shown in solid gray, and connections specific to the outlier network in dashed red. The shift centrality of TNF-α and Tnfrsf1b is evident.

A comparative analysis of node centrality (measured by degree) revealed a key redistribution in the relative importance of markers (Figure 13, Table 15). TNF-α emerged as a hub in the outlier network. While on the normal network TNF-α had no significant connections (centrality = 0), in the outlier network it showed the highest centrality (degree = 1, relative centrality = 0.200), establishing a key connection. In contrast, Tnfrsf1b reduced its central role; although it functioned as a connected node in the normal network (centrality = 0.200), it lost all its significant connections in the outlier network (centrality = 0).

This visualization highlights how the co-expression network is reconfigured in the outlier state: TNF-α acquires a central a connective role, while Tnfrsf1b becomes disconnected from the main network. This suggests a shift in the predominant signaling axes, where the effector cytokine TNF-α gains prominence in coordinating the extreme inflammatory state, potentially at the expense of regulatory pathways mediated by its specific receptor, Tnfrsf1b.

### 3.14. Unsupervised Clustering Analysis for Identification of In Vitro Inflammatory Sub-Phenotypes

#### 3.14.1. Evaluation of Clustering Tendency in the *In Vitro* Model

Prior to applying clustering algorithms, the clustering tendency of the data was rigorously evaluated to determine if an underlying structure justified the search for subpopulations. This analysis employed a multimodal approach combining statistical metrics and visual assessment.

First, the Hopkins statistic was calculated, a measure that compares the distance distribution in the real dataset with a uniformly random dataset of the same dimensional space. A value of 0.5 indicates randomness, while values close to 1 suggest a strong clustering tendency. For our gene expression dataset (n = 39, 6 variables), the Hopkins statistic was 0.7865, indicating a moderate to strong clustering tendency, justifying the search for substructures.

**Figure 14:**
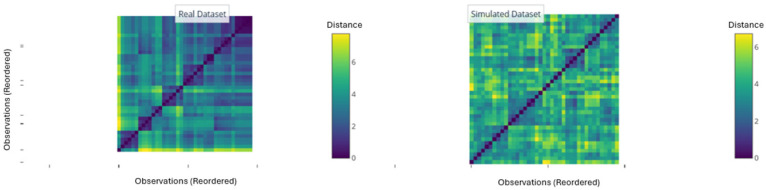
Evaluation of clustering tendency (VAT). Reordered Euclidean distance matrices for the real dataset (**left panel**) and a simulated random dataset (**right panel**). The presence of dark square blocks along the diagonal in the real dataset is a diagnostic pattern indicating the existence of natural, compact clusters. The absence of this pattern in the simulated dataset confirms that the observed structure in the experimental data is not random.

Subsequently, reordered Euclidean distance matrices (Visual Assessment of Cluster Tendency, VAT) were generated for the real dataset and for a simulated dataset without structure. The matrix of the real dataset showed the formation of dark blocks along the diagonal, a typical pattern indicating the existence of compact groups. In contrast, the matrix of the simulated dataset presented a homogeneous random pattern with no defined blocks (Figure 14).

While the simulated data were uniformly distributed and structureless, the real dataset showed an arrangement with discernible groupings, visually corroborating the signal detected by the Hopkins statistics and the VAT analysis.

#### 3.14.2. Determination of the Optimal Number of Clusters

With clustering tendency validated, the optimal number of clusters (k) was determined using widely recognized internal validation indices: the Elbow method (based on the Within-Cluster Sum of Squares, WSS), the Silhouette Coefficient, the Calinski–Harabasz index (CH), and the Davies–Bouldin index (DB). The analysis was applied to the standardized matrix of the six expression markers (IL-10, ADIPOQ, IL-6, Tnfrsf1a, Tnfrsf1b), considering a range of k from two to six clusters.

The convergence of the four validation methods unanimously supported the selection of k = 2 as the optimal number of clusters for this dataset (Table 16). In the WSS method, the reduction in Within-Cluster Sum of Squares showed an inflection point (elbow) at k = 2. The Silhouette Coefficient reached its maximum value (0.6471) at k = 2, indicating a strong, well-defined cluster structure where most observations were well-matched with their assigned cluster. The CH method presented its highest value (17.96) at k = 2, reflecting the best balance between inter-cluster separation and intra-cluster cohesion. The DB index reached its minimum value (0.3928) at k = 2, denoting a low average similarity between clusters (high distinction between them).

The consistency and robustness of this convergence across diverse quantitative and qualitative methods provide a solid foundation for proceeding with the K-means clustering algorithm using k = 2.

#### 3.14.3. Application of the K-Means Algorithm and Cluster Characterization

With the optimal number of clusters established as k = 2, the K-means algorithm was applied to the standardized matrix of the six gene expression markers. The quality of the partition was validated using performance indices, showing a robust clustering structure: a Silhouette Coefficient of 0.647 (indicating a strong structure), a CH index of 18.0, and a DB index of 0.393.

The partition identified two clusters of distinct sizes: Cluster 0 (n = 3, 60%) and Cluster 1 (n = 2, 40%). A critical finding was that these two clusters were composed exclusively of the five observations pre-identified as outliers, confirming that the algorithm captured heterogeneity within these extreme observations.

#### 3.14.4. Validation via Agglomerative Hierarchical Clustering

To confirm the robustness and nature structure of the partition identified by K-means, a complementary and independent clustering approach was applied: agglomerative hierarchical clustering. A dendrogram was constructed using Euclidean distance and the complete linkage method representing the hierarchy of similarity among all observations.

**Figure 15:**
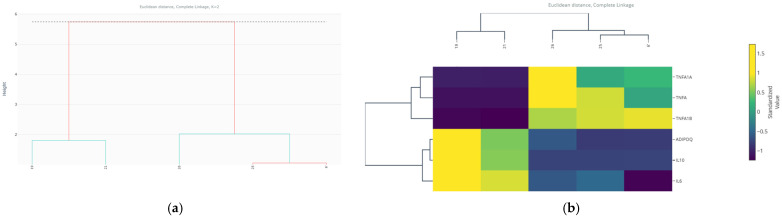
Hierarchical clustering analysis and characterization of cluster sub-phenotypes. (**a**) Dendrogram of agglomerative hierarchical clustering (Euclidean distance, complete linkage). The red horizontal line indicates the cut to obtain k = 2 clusters. The colored branches correspond to the two identified groups. (**b**) Coupled heatmap showing the standardized expression profiles (Z-score) of the six markers for the five outlier observations, ordered according to the dendrogram.

The resulting dendrogram (Figure 15a) showed a fundamental primary division that clearly separated the 39 observations into two main branches. Cutting the dendrogram at the height corresponding to k = 2 produced a cluster assignment identical to that obtained by K-means, independently validating the existence of two discrete groups. This replication by a different algorithmic method underscores the robustness of the underlying bimodal structure in the data.

Inspection of the heatmap coupled to the dendrogram (Figure 15b), which shows the standardized expression values for the six markers, allowed characterization of the distinct molecular profiles of each sub-phenotype.

First, Cluster 0/Branch 0 (IDs: 21, 25, 26) was characterized by a profile of coordinated hyper-expression of IL-6 and Tnfrsf1a, alongside moderate expression of TNF-α. Simultaneously, it showed marked expression of the anti-inflammatory cytokine IL-10.

Second, Cluster 1/Branch 1 (IDs: 6, 19) exhibited an almost opposite pattern: very low expression of IL-6 and Tnfrsf1a, along with null or low levels of TNF-α. However, it shared with Cluster 1 the strong suppression of IL-10.

**Figure 16:**
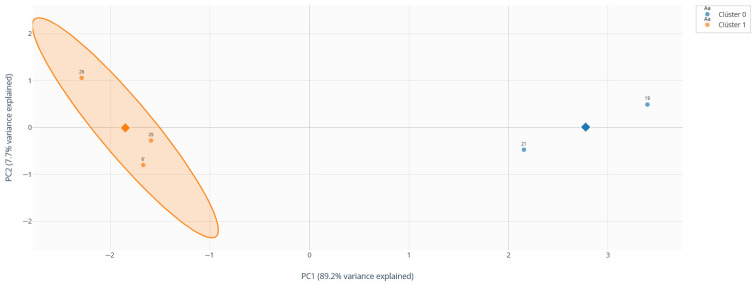
Hierarchical biplot (Euclidean distance, complete linkage method). PCA projection (PC1 and PC2) of observations colored according to their hierarchical cluster assignment (Cluster 0: blue, dark blue diamond symbol represents the cluster centroid; Cluster 1: orange, dark orange diamond symbol represents the cluster centroid). Sample IDs are shown next to the points.

To visualize the spatial relationship of these subgroups within the multivariate context, a hierarchical biplot was generated (Figure 16). In this visualization, observations (labeled with their IDs) are colored according to their dendrogram assignment and overlaid with the marker vectors. This figure confirms that: (1) the two clusters occupy extreme and opposite regions in the PC1 space; (2) the position of the Cluster 0 is strongly associated with the IL-6 and Tnfrsf1a vectors; (3) Cluster 1 is in the opposite region, away from these pro-inflammatory vectors.

The convergence of K-means and hierarchical clustering methods robustly identifies two discrete inflammatory sub-phenotypes within the outliers of the 3T3-L1 model: first, a classical pro-inflammatory phenotype (Cluster 0), defined by the extreme co-activation of IL-6/Tnfrsf1a; second, an atypical non-hyperinflammatory phenotype (Cluster 1), which lacks this pro-inflammatory pattern but shares the profound suppression of IL-10.

This finding demonstrates that the outlier condition is not homogeneous and can arise from different molecular imbalances. The common suppression of IL-10 in both groups suggests that the loss of anti-inflammatory regulation could be a central and shared pathogenic mechanism that predisposes the cell to extreme states, whether towards hyperinflammation (Cluster 0) or towards another form of dysfunction (Cluster 1).

### 3.15. Considerations on Predictive Modeling in the Context of the In Vitro Model

The final step in the analytical framework applied to this in vitro cohort consisted of constructing a Random Forest predictive model to identify the key determinants of the outlier phenotype. However, the direct application of this approach to the in vitro dataset faces a fundamental methodological limitation from sample size.

While the outlier subpopulation in the diabetic neuropathy cohort (n = 44) allowed for robust model training and validation, the reduced number of outliers in the 3T3-L1 model (n = 5) precludes an analogous predictive analysis with statistical integrity. With a minority class of only five observations, any supervised machine learning algorithm, including Random Forest, would be subject to extreme overfitting. In such a scenario, the model would learn the idiosyncrasies of those few samples rather than generalizable patterns, and performance metrics (area under the ROC curve) and variables’ importance would completely lack stability, statistical power, and external validity.

Therefore, in the interest of methodological rigor, the application of a supervised classification model to this dataset was refrained from. Instead, the analysis focused on maximizing extractable knowledge through rigorous univariate and bivariate characterization (descriptive statistics, Mann U tests, association analysis with categorical variables); multivariate structure analysis (PCA, co-expression network analysis to elucidate relationships between markers); and unsupervised clustering to deconvolute the internal heterogeneity of the outliers and propose discrete biological sub-phenotypes.

This strategy ensures that inferences derived from the in vitro model are based on methods that are robust and appropriate for its scale, avoiding spurious conclusions. The validation of the identified extreme profiles (especially the two sub-phenotypes revealed by clustering) and the investigation of their underlying causes constitute a clear objective for future studies, which should be designed with a replicative scale that permits reliable predictive analyses.

The cross-sectional application of the analytical framework to two dissimilar biological systems (in vitro and clinical) validates the generalizable power of the approach. The robust identification of extreme profiles through multi-algorithm consensus, followed by multivariate, network, and unsupervised characterization, demonstrates that outliers represent discrete biological entities with specific underlying mechanisms—whether a canalized hyperactivation of Th1/Th17 pathways in patients or an extreme dysregulation of the IL-6/IL-10 axis in adipocytes. This consistency corroborates that the systematic investigation of atypical observations transcends methodological control to become a discovery strategy: it reveals critical pathophysiological heterogeneity, defines molecular subtypes, and prioritizes mechanistic targets that average-focused analyses obscure.

## 4. Discussion

Previous studies have demonstrated that extreme gene expression patterns can reveal biologically meaningful signals, particularly in cancer genomics and rare disease diagnostics. Methods such as COPA [18] and more recent RNA-seq-based frameworks like OUTRIDER [19] have successfully leveraged expression outliers to detect oncogenic rearrangements or pathogenic transcriptional alterations. However, these approaches primarily focus on identifying anomalous expression values at the gene level. In contrast, the framework proposed in this study extends the concept of outlier analysis beyond detection by integrating consensus-based anomaly identification with multivariate characterization, network analysis, unsupervised stratification, and predictive modeling. This multi-layer analytical strategy enables the interpretation of extreme observations as coherent immunometabolic phenotypes rather than isolated statistical anomalies, thereby providing an additional perspective for investigating biological heterogeneity in complex diseases.

Our study implements a robust and reproducible analytical framework that transcends traditional univariate approaches, integrating multimodal detection, multivariate characterization, and predictive modeling. This strategy allowed us not only to identify extreme profiles but also to unravel their molecular and clinical architecture, revealing discrete molecular subtypes within metabolic–inflammatory syndromes.

The systematic application of an outlier detection and characterization framework to two distinct biological systems reveals the existence of discrete pathobiological entities defined by specific molecular mechanisms. This study empirically validates the conceptual premise that extreme observations in biomedical data do not constitute experimental noise but rather signals of high-consequence biological phenomena—a notion historically supported by seminal cases such as the discovery of Kawasaki disease or the pharmacogenetics of statin-induced rhabdomyolysis [35,36]. Our novel methodological approach, which transcends classical univariate strategies through a robust multi-algorithm consensus, detects, characterizes, and interprets these extreme profiles, positioning itself as a tool for the discovery of disease subtypes.

An aspect that warrants consideration is the relatively high proportion of individuals classified as outliers in the clinical cohort (47.3%). In classical statistical settings, outliers are typically expected to represent rare observations. However, in high-dimensional biological systems, multivariate anomaly detection often identifies broader subsets of samples that diverge from the dominant population structure rather than from individual variables. In transcriptomic studies, similar patterns have been described where extreme expression signatures revealed biologically meaningful molecular subgroups rather than isolated statistical analysis [37]. Importantly, the consensus-based strategy used in this study was specifically designed to balance sensitivity and specificity by requiring confirmation across multiple independent detection methods, thereby reducing the risk of positive false classifications that could arise from relying on a single algorithm.

The bootstrap stability analysis further supports the reliability of the detected extreme profiles. Although the stability values were moderate, this behavior is expected in high-dimensional biological datasets where multivariate anomaly detection identifies samples that deviate from the dominant population structure rather than isolated statistical analysis. Similar stability patterns have been reported in anomaly detection frameworks applied to complex biomedical data, where bootstrap resampling often reveals partial but reproducible detection of atypical observations due to intrinsic biological heterogeneity [38].

In the diabetic neuropathy cohort, the extreme immunometabolic profiles (47.3%) were characterized by coordinated and disproportionate hyperactivation of the Th1/Th17 axis, with extreme elevations in T-bet and IL-17, and concurrent dyslipidemia, specifically hypertriglyceridemia. This pattern suggests a fundamental immune reprogramming that exceeds the typical low-grade inflammation of diabetes. The specificity of this dysregulation is crucial: while TNF-α showed no change, the IL-12/STAT4/T-bet pathway, which drives Th1 differentiation [39], emerged as central. This aligns with evidence linking effector T-cell activation, particularly that of Th17 cells, to the pathogenesis of neuropathic pain and axonal damage in diabetes models, where neutralization of IL-17 or IL-23 attenuates neuropathy [40,41]. Our findings extend these observations to the human clinical context, proposing that a patient subgroup exhibits specific adaptive neuroinflammation, possibly driven by autoantigens or damage-associated molecular patterns in the neural microenvironment. The emergence of TGFβ as the sole central hub in the outlier co-expression network links this immune hyperactivation to fibrosis and tissue remodeling pathways, which are central to the progression of diabetic neuropathy toward irreversible axonal degeneration [42].

The co-occurrence of extreme hypertriglyceridemia is unlikely to be incidental but suggests an integrated pathophysiological circuit. Triglycerides and free fatty acids can act as ligands for innate immunity receptors (TLR2/4) on macrophages and Schwann cells, amplifying the production of pro-inflammatory cytokines like IL-1β and IL-6 via NLRP3 inflammasome activation [43,44]. Thus, dyslipidemia could directly potentiate the observed Th1/Th17 immunological phenotype, creating a positive feedback loop that exacerbates cellular metabolic stress and neuronal damage. This model of immune–metabolic interaction is consistent with studies that identify specific lipid patterns associated with an increased risk of microvascular complications, independent of glycemic control [45].

Furthermore, the identification of sub-phenotypes within the outlier population itself, through unsupervised clustering, underscores the underlying biological heterogeneity and reinforces the utility of this approach for patient stratification. This aligns with contemporary precision medicine initiatives, where deep phenotyping has revealed clinically meaningful subgroups with distinct molecular drivers and outcomes in complex diseases such as diabetes and immune-mediated disorders [46]. This level of granularity could inform personalized therapeutic strategies specific pathways, such as TGFβ signaling or the IL-6/IL-10 axis.

The identification of sub-phenotypes within the outliers via unsupervised clustering- one with a global hyperactivation profile and another more canalized towards TGFβ- reveals stratified heterogeneity within atypicality. This diversity suggests multiple breakpoints in the regulatory network, possibly with distinct genetic substrates (polymorphisms in JAK-STAT or TGFβ signaling pathway genes) or environmental exposures. The predictive capacity of the Random Forest model (AUC > 0.78), integrating renal function (creatinine) and metabolic variables with immunological markers, reinforces the multimodal nature of the phenotype and suggests a potential translational pattern for risk stratification, in line with precision medicine initiatives in diabetes [47].

The application of the same framework to the in vitro 3T3-L1 adipocyte model provides mechanistic validation in a reductionist system. The cellular outliers (12.8%), characterized by massive IL-6 production and profound suppression of IL-10, represent an autonomous and extreme pro-inflammatory state. IL-6 is a pleiotropic cytokine that, when chronically elevated, induces insulin resistance, endothelial dysfunction, and nociceptive sensitization, thereby directly linking it to the pathogenesis of neuropathy [48]. The concurrent suppression of IL-10, a potent anti-inflammatory regulator, suggests a critical failure in resolution mechanisms, allowing the perpetuation of inflammatory signaling. This finding is consistent with studies reporting a lower secretory capacity for IL-10 in monocytes from patients with diabetes and neuropathy [49]. Network analysis showed a reconfiguration in this state, where TNF-α gained centrality at the expense of its receptor Tnfrsf1b, indicating a possible shift in signaling balance towards feedback pathways mediated by the soluble cytokine.

The consistency of findings across systems, the identification of subpopulations with specific cytokine dysregulation (IL-17/IL-6) and loss of negative regulation (IL-10), points to a general principle: outliers materialize extreme deviations at critical nodes of homeostatic networks. Although the specific involved differs between the clinical cohort and the adipocyte model, both systems converge on dysregulated inflammatory signaling, supporting the robustness of the analytical framework rather than strict molecular replication across experimental contexts. This approach is analogous to intentional discovery strategies like the study of extreme phenotypes in human genetics or knockout models, where maximal perturbation illuminates physiological function [50]. Here, the “perturbation” is emergent, either naturally or experimentally, and our framework detects it *a posteriori* in complex data.

From a methodological perspective, this study demonstrates the superiority of multivariate, density-based anomaly detection algorithms (LOF, Isolation Forest) for capturing complex profiles that would be invisible to univariate methods or standard PCA, which is notoriously sensitive to and can be distorted by outliers [51,52,53]. Our adaptive consensus mitigates the limitations of individual methods, offering a reproducible analytical framework for anomaly detection in biomedicine, an emerging field that promises to accelerate discovery by prioritizing the exceptional for in-depth investigation.

### Limitations and Future Perspectives

The main limitations include the cross-sectional nature of the clinical cohort and the limited size of the outlier subgroups, particularly in the in vitro model (n = 5), which limits causal inferences and statistical generalization. Validation in independent, large-scale longitudinal cohorts is essential to confirm the prognostic value of the identified phenotype. Future studies should integrate multi-omics data to elucidate the genetic and epigenetic determinants of this extreme susceptibility. Meanwhile, in experimental models, the manipulation of priority candidates (the IL-6/IL-10 and TGFβ pathways) will allow testing of their causal role. Applying this framework to other complex diseases will assess its general utility for discovering hidden molecular subtypes.

## 5. Conclusions

This study demonstrates that the systematic application of a robust analytical framework for the detection and characterization of outliers constitutes a transformative strategy for translational biomedical research. By shifting from the paradigm of their removal to that of their active investigation, through a multi-algorithm consensus, network analysis, unsupervised clustering, and predictive modeling, we have identified and characterized biologically coherent extreme phenotypes in two distinct systems: a subgroup of patients with diabetic neuropathy exhibiting a hyperactivated, specific immune–metabolic profile (defined by the Th1/Th17 pathway and extreme dyslipidemia), and a subset of adipocytes with autonomous, dysregulated pro-inflammatory state (characterized by hyperproduction of IL-6 and suppression of IL-10).

The findings corroborate that outliers are not methodological artifacts, but discrete entities that reveal tipping points within complex homeostatic networks. Their study enables: (1) the discovery of disease subtypes not captured by averaged analyses; (2) the prioritization of key molecular mechanisms (STAT4 signaling, IL-6/IL-10 imbalance); (3) the proposal of integrated patterns for patient stratification and the identification of specific therapeutic targets.

Beyond the specific context of metabolic–inflammatory syndromes, this work establishes a generalizable, reproducible methodological pipeline that operationalizes anomaly science in biomedicine. The rigorous investigation of the most extreme profiles thus emerges as a powerful avenue to accelerate the discovery of novel mechanisms and lay the groundwork for precision medicine truly driven by underlying biological heterogeneity.

## Figures and Tables

**Table 1:** Distribution of participants by intervention group and assessment time point.

Time Point	Electroacupuncture (n)	Control (n)	Total (n)
Basal	16	15	31
ME02	16	15	31
ME03	16	15	31
Total	48	45	93

**Table 2:** Distribution of categorical variables between outliers (n = 44) and normal data (n = 49) groups.

Variable	Category	Outliers n (%)	Normal Data n (%)
Group	Intervention	24 (54.2%)	21 (42.9%)
	No intervention	20 (45.8%)	28 (57.1%)
Time point	Basal	14 (33.3%)	17 (33.3%)
	ME02	14 (33.3%)	17 (33.3%)
	ME03	14 (33.3%)	17 (33.3%)

**Table 3:** Descriptive statistics of immunological mediator expression (normalized relative units).

Marker	Group	Mean ± SD *	Median (IQR *)	Range (Min–Max)
IL-17	Outliers	82.89 ± 292.22	3.95 (0.83–29.42)	0.0018–1944.06
	Normal	4.03 ± 6.20	1.95 (0.68–4.61)	0.0271–25.37
T-bet	Outliers	44.62 ± 155.57	3.22 (0.83–21.30)	0.0060–1216.8
	Normal	1.32 ± 2.00	0.40 (0.12–1.35)	0.0365–7.94
RORγT	Outliers	32.31 ± 80.61	2.40 (0.85–21.30)	0.0178–445.68
	Normal	5.45 ± 9.52	1.81 (0.26–7.37)	0.0331–33.64
SOCS3	Outliers	220.27± 1619.36	3.94 (0.50–18.67)	0.0088–13,759.52
	Normal	2.59 ± 4.13	0.41 (0.10–3.42)	0.0107–16.34
STAT4	Outliers	56.05 ± 213.64	3.26 (0.88–32.51)	0.0022–1422.83
	Normal	2.95 ± 6.72	0.60 (0.11–1.74)	0.0283–28.37
TNF-α	Outliers	495.63± 1842.28	3.50 (0.07–194.54)	0.0000–11,439.05
	Normal	38.56 ± 74.81	2.29 (0.01–17.72)	0.0000–229.82

* SD: Standard Deviation; IQR: Interquartile Range.

**Table 4:** Association between outlier status and categorical variables.

Variable	Chi-Square Statistic (χ^2^)	Degrees of Freedom	*p*-Value	Effect Size	Significance
Intervention	0.441	1	0.507	φ = 0.069 (small)	Non-significant
Time Point	0.0000	2	1.000	V = 0.000 (small)	Non-significant

**Table 5:** Comparison of immunological and metabolic markers between outlier and normal groups using the Mann–Whitney U test.

Variable	U Statistic	*p*-Value	Effect Size (r)	Significance
T-bet	1144.0	0.00037 ***	0.37 (medium)	Significant
TGFβ	1106.0	0.0013 **	0.33 (medium)	Significant
SOCS3	1051.0	0.0068 **	0.28 (small–medium)	Significant
STAT5	1011.5	0.019 *	0.24 (small–medium)	Significant
SOCS1	1014.0	0.018 *	0.25 (small–medium)	Significant
STAT3	1003.0	0.024 *	0.24 (small–medium)	Significant
STAT4	968.0	0.052.	0.20 (small–medium)	Marginal/Borderline
STAT6	954.0	0.070	0.19 (small–medium)	Non-Significant
IL-17	933.0	0.17	0.17 (small–medium)	Non-Significant
RORγT	865.0	0.32	0.10 (small)	Non-Significant
TNF-α	820.0	0.56	0.06 (small)	Non-Significant

*** *p* < 0.001; ** *p* < 0.01; * *p* < 0.05; *p* < 0.1. r: Rosenthal’s r.

**Table 6:** Variance explained by principal components of the immunological profile in diabetic neuropathy.

Component	Explained Variance (%)	Cumulative Variance (%)
PC1	47.6	47.6
PC2	10.2	57.8
PC3	9.5	67.3
PC4	7.5	74.8
PC5	6.6	81.4

**Table 7:** Major changes in node centrality between the co-expression network of the outlier and normal groups.

Variable	Centrality (Outlier)	Centrality (Normal)	Δ Centrality	Insights
TGFβ	0.444	0.056	+0.389	Emerges as a new principal hub in outliers
STAT4	0.444	0.167	+0.278	Gains central importance in the atypical network
SOCS3	0.500	0.278	+0.222	Increases its connectivity
STAT5	0.333	0.111	+0.222	Gains relevance as a connected node
T-bet	0.389	0.222	+0.167	Increases its connectivity
STAT6	0.444	0.278	+0.167	Greater centrality
SOCS1	0.500	0.333	+0.167	Remains a hub but gains strength
Uric Acid	0.000	0.222	−0.222	Loses its hub status completely
Glucose	0.000	0.222	−0.222	Disconnects from the main network

**Table 8:** Characterization of clusters identified by unsupervised clustering within the outlier subgroup (n = 44).

Cluster	Size (n)	Percentage	Position in PCA (PC1, PC2)	Composition
0 (Minority)	3	6.8%	(8.291, 0.636)	Extreme outlier subset (IDs: A-GGG-24, A-RJM-11, A-GMG-23 *)
1 (Majority)	41	93.2%	(−0.607, −0.047)	Core outlier subgroup

* Representative IDs from hierarchical clustering dendrogram.

**Table 9:** Performance metrics of the Random Forest model for predicting the outlier phenotype on the test set.

Metric	Value	Insights
AUC-ROC	0.783	Good discriminative ability
Accuracy	0.842	84.25 of predictions are correct
Positive Predictive Value (Precision)	0.833	83.35 of those predicted as outliers are true outliers
Sensitivity (Recall)	1.000	100% detection of true outliers
Specificity	0.714	Correct classification of 71.4% of normal individuals

**Table 10:** Relative importance of predictor variables in the Random Forest model (Top 10).

Variable	Importance (Gini *)	Percentage (%)	Domain
Creatinine	0.1063	10.63%	Biochemical
T-bet	0.1042	10.42%	Immunological
Uric Acid	0.0764	7.64%	Biochemical
STAT5	0.0712	7.12%	Immunological
SOCS3	0.0700	7.00%	Immunological
SOCS1	0.0681	6.81%	Immunological
TGFβ	0.0664	6.64%	Immunological
STAT4	0.0584	5.84%	Immunological
BUN	0.0535	5.35%	Biochemical
Glucose	0.0524	5.24%	Metabolic

* The Gini importance values represent the mean decrease in node impurity across all trees. Percentages are calculated relative to the total importance sum of the top 10 predictors.

**Table 11:** Descriptive distribution of outliers according to the experimental conditions of the in vitro study.

Variable	Category	Outliers (n = 5)	Normal Data (n = 34)	Total Per Category
Group	Basal	3 (60.0%)	12 (35	Basal
	siRNA Tnfrsf1b	2 (40.0%)	10 (29.4%)	12
	SiRNA Tnfrsf1a	0 (0.0%)	12 (35.3%)	12
Treatment	TNF-α	2 (40.0%)	1 (2.9%)	3
	Control	2 (40.0%)	7 (20.6%)	9
	TNF-α + Glycine	1 (20.0%)	8 (23.5%)	9
	Glycine	0 (0.0%)	9 (26.5%)	9
	Glycine + TNF-α	0 (0.0%)	9 (26.5%)	9

**Table 12:** Association between outlier status and experimental categorical variables.

Variable	Statistic	Value	Degrees of Freedom	*p*-Value	Effect Size (Cramer’s V)
Group	χ^2^	2.62	2	0.796	0.26
	χ^2^	11.16	4	0.152	0.54

Chi-square test with Monte Carlo simulation (10,000 replicates).

**Table 13:** Comparison of gene expression markers between outlier and normal groups in the 3T3-L1 model.

Variable	Outlier Group Median (IQR)	Normal Group Median (IQR)	U Statistic	*p*-Value	Effect Size (r)
Il-6	6.92 (3.71–15.56)	0.03 (0.00–0.58)	159.0	0.002 *	0.50
Tnfrsf1a	2.82 (0.03–6.60)	0.07 (0.02–0.32)	117.0	0.186	0.22
IL-10	0.003 (0.002–1.46)	1.47 (0.26–7.94)	45.0	0.097	−0.27
Tnfrsf1b	0.32 (0.03–0.36)	0.31 (0.10–0.82)	56.5	0.239	−0.19
TNF-α	2.62 (0.00–5.39)	0.08 (0.01–1.35)	104.0	0.434	0.13
ADIPOQ	0.77 (0.50–3.61)	1.56 (1.00–2.32)	72.0	0.600	−0.09

IQR: Interquartile Range; r: Rosenthal’s effect size (|r| ≥ 0.5: large; 0.3–0.5: medium; 0.1–0.3: small to medium; <0.1: small). * *p* < 0.05.

**Table 14:** Variance explained by the principal components of the gene expression profile in 3T3-L1 adipocytes.

Component	Explained Variance (%)	Cumulative Variance (%)
PC1	34.9	34.9
PC2	24.0	58.9
PC3	16.5	75.5
PC4	14.0	89.5
PC5	6.1	95.6
PC6	4.4	100.00

**Table 15:** Changes in node centrality between the co-expression networks of the normal and outlier groups.

Variable	Centrality (Outliers)	Centrality (Normals)	Δ Centrality	Insights
TNF-α	0.200	0.000	+0.200	Emerges as a new hub in outliers
Tnfrsf1b	0.000	0.200	−0.200	Loses its hub status in outliers
Other markers	0.000	0.000	0.000	No change in connectivity

Δ: change in centrality between outlier and normal groups.

**Table 16:** Values of validation indices for determining the optimal number of clusters (k). The optimal value for each index is highlighted in bold. For the Silhouette Coefficient and the Calinski–Harabasz index, higher values indicate a better clustering structure. For the Davies–Bouldin index, a lower value indicates better separation between clusters.

K	Within-Cluster Sum of Squares (WSS)	Silhouette Coefficient	Calinski–Harabasz Index	Davies–Bouldin Index
2	4.29	0.6471	17.96	0.3928
3	2.82	0.4922	15.23	0.6214
4	2.02	0.3645	15.07	0.8123
5	1.36	0.3011	16.58	0.8701
6	0.94	0.2214	18.76	1.0657

## Data Availability

The processed datasets generated and analyzed during this study, specifically the gene expression matrix from the 3T3-L1 adipocyte experiment and the integrated baseline dataset from the diabetic neuropathy clinical cohort, are available in a publicly accessible repository. They can be downloaded via de following link: https://drive.google.com/drive/folders/1Y5N7oYjx6oMf9eZDHEewztqitbQaVyZW?usp=sharing. All custom computer code and scripts for the analytical pipeline, including the multi-algorithm consensus framework, statistical analysis, and web application, are publicly available in the GitHub repository: https://github.com/jjhav88/Outlier-Detection-Framework.

## Appendix A

**Table A1:** Descriptive statistics of total cholesterol and triglyceride levels in the outlier and normal groups.

Lipid Parameter	Statistic	Outlier (n = 44)	Normal (n = 49)
Total cholesterol	Mean	175.4	170.3
	Median	171.5	186.0
	Standard Deviation	36.5	49.8
	Minimum	106.0	88.0
	Maximum	303.0	257.0
Triglycerides	Mean	197.9	166.8
	Median	159.5	146.6
	Standard Deviation	134.8	66.6
	Minimum	12.5	82.7
	Maximum	834.3	327.3

The median triglyceride level in the outlier group was 1.6 times higher than that in the normal group. The maximum triglyceride value in outliers exceeded that of the normal group by a factor of 2.5, reflecting extreme elevation and high variability consistent with immunological markers described in the main text.
